# Supplementary material for: Optimizing network neuroscience computation of individual differences in human spontaneous brain activity for test-retest reliability
Source: Netw Neurosci. 2023 Oct 1;7(3):1080–108. doi: 10.1162/netn_a_00315 (PMC10473278; doi:10.1162/netn_a_00315)
Supplement: Supplementary file 1 [file netn-7-3-1080-s001.pdf]

Supplementary Information

Optimizing network neuroscience computation of individual differences in human spontaneous brain activity for test-retest reliability

Contents

List of Figures 2

List of Tables 3

1 Parcellations - Node Definition 5
1.1 ICC Density distribution . . . 5
1.2 ICC Almost Perfect (ICCs > 0.8) . . . 6
1.3 Substantial or Above (ICCs > 0.6) . . . 7
1.4 Descriptive statistics Mean . . . 9
1.5 Descriptive statistics Median . . . 10
1.6 Friedman Test . . . 10
1.7 Friedman Test Effect size . . . 10
1.8 Paired Wilcoxon signed rank test . . . 11
1.9 Kullback-leibler divergence Map . . . 18
1.10 Significance Map . . . 19
1.11 Effect size . . . 19
1.12 Variability Changes . . . 26

2 Frequency Bands - Edge Construction 28
2.1 ICC Density distribution . . . 28
2.2 Almost Perfect (ICCs > 0.8) . . . 28
2.3 Substantial or Above (ICCs > 0.6) . . . 29
2.4 Variability Changes . . . 30
2.5 Descriptive statistics Mean . . . 31
2.6 Descriptive statistics Median . . . 31
2.7 Friedman Test . . . 32
2.8 Friedman Test Effect size . . . 32
2.9 Paired Wilcoxon signed rank test . . . 32
2.10 Kullback-leibler divergence Map . . . 33
2.11 Significance Map . . . 33
2.12 Effect size . . . 33

3 R Tranforms - Edge Construction 35
3.1 ICC Density distribution . . . 36
3.2 Almost Perfect (ICCs > 0.8) . . . 36
3.3 Substantial or Above (ICCs > 0.6) . . . 37
3.4 Descriptive statistics Mean . . . 37
3.5 Descriptive statistics Median . . . 38
3.6 Friedman Test . . . 38
3.7 Friedman Test Effect size . . . 38
3.8 Paired Wilcoxon signed rank test . . . 38
3.9 Kullback-leibler divergence Map . . . 39

3.10 Significance map 39
3.11 Effect size 39

4 Schemes - Edge Construction **40**
4.1 ICC Density distribution 40
4.2 Almost Perfect (ICCs > 0.8) 41
4.3 Substantial or Above (ICCs > 0.6) 42
4.4 Varibility Changes 42
4.5 Descriptive statistics Mean 44
4.6 Descriptive statistics Median 45
4.7 Friedman Test 45
4.8 Friedman Test Effect size 45
4.9 Paired Wilcoxon signed rank test 45
4.10 Kullback-leibler divergence Map 47
4.11 Significance map 48
4.12 Effect size 48

5 Metrics - Network Analysis **50**
5.1 Metrics 50
5.2 ICC Density distribution 52
5.3 Almost Perfect (ICCs > 0.8) 52
5.4 Substantial or Above (ICCs > 0.6) 53
5.5 Descriptive statistics Mean 54
5.6 Descriptive statistics Median 54
5.7 Paired Wilcoxon signed rank test 54
5.8 Kullback-leibler divergence Map 55
5.9 Significance map 56
5.10 Effect size 56

6 More Metrics - Network Analysis **58**
6.1 ICC Density distribution 58
6.2 Almost Perfect (ICCs > 0.8) 59
6.3 Substantial or Above (ICCs > 0.6) 60
6.4 Descriptive statistics Mean 62
6.5 Descriptive statistics Median 64
6.6 Kullback-leibler divergence Map 66
6.7 Significance map 67

7 fMRIPrep pipeline **67**

References **71**

List of Figures

S1 Parcellation - Density distribution 5
S2 Parcellation - Number of ICCs > 0.8 6
S3 Parcellation - Number of ICCs > 0.6 8
S4 Parcellation - KL divergence Map 18
S5 Parcellation - Significance Map 19
S6 Parcellation - Effect size 26
S7 Parcellation - Variability Changes 27
S8 Frequency - ICC Density distribution 28
S9 Frequency - Number of ICC >0.8 28
S10 Frequency - Number of ICC >0.6 29

S11 Frequency - Variability Changes 30
S12 Frequency - ICC Mean Bar plot 31
S13 Frequency - KL divergence Map 33
S14 Frequency - Significance Map 33
S15 Frequency - Effect size 34
S16 Transform r to weight 35
S17 Transforms - ICC Density distribution 36
S18 Transforms - Number of ICC > 0.8 36
S19 Transforms - Number of ICC > 0.8 37
S20 Transforms - KL divergence Map 39
S21 Transforms - Significance Map 39
S22 Transforms - Effect size 40
S23 Schemes - ICC Density distribution 40
S24 Schemes - Number of ICC > 0.8 41
S25 Schemes - Number of ICC > 0.6 42
S26 Schemes - Variability Changes 43
S27 Schemes - ICC mean and se 44
S28 Schemes - KL divergence Map 47
S29 Schemes - Significance Map 48
S30 Schemes - Effect size 50
S31 Metrics - Density distribution 52
S32 Metrics - Number of ICC > 0.8 53
S33 Metrics - Number of ICC > 0.6 54
S34 Metrics - KL divergence Map 55
S35 Metrics - Significance Map 56
S36 Metrics - Effect size 56
S37 Metrics - Density distribution 58
S38 Metrics - Number of ICC > 0.8 60
S39 Metrics - Number of ICC > 0.6 62
S40 Metrics - KL divergence Map 66
S41 Metrics - Significance Map 67
S42 Test-retest reliability of fMRIPrep-preprocessed data 68
S43 Reliability anatomy of fMRIPrep-preprocessed data 69

List of Tables

S1 Parcellation - Number of ICCs > 0.8 6
S2 Parcellation - Number of ICCs > 0.6 7
S3 Parcellation - ICC Mean 9
S4 Parcellation - ICC Median 10
S5 Parcellation - Friedman Test 10
S6 Parcellation - Friedman Test Effect size 10
S7 Parcellation - Paired Wilcoxon signed rank test 11
S8 Parcellation - Effect size 19
S9 Frequency - ICC Mean 31
S10 Frequency - ICC Median 31
S11 Frequency - Friedman Test 32
S12 Frequency - Friedman Test Effect size 32
S13 Frequency - ICC group1 vs. group2 32
S14 Frequency - Effect size 34
S15 Transforms - Number of ICCs > 0.8 36
S16 Transforms - Number of ICCs > 0.6 37
S17 Transforms - ICC Mean 37

S18 Transforms - ICC Median 38
S19 Transforms - Friedman Test 38
S20 Transforms - Friedman Test Effect size 38
S21 Transforms - ICC group1 vs. group2 38
S22 Transforms - Effect size 39
S23 Schemes - Number of ICCs > 0.8 41
S24 Schemes - Number of ICCs > 0.8 42
S25 Schemes - ICC Mean 44
S26 Schemes - ICC Median 45
S27 Schemes - Friedman Test 45
S28 Schemes - Friedman Test Effect size 45
S29 Schemes - ICC group1 vs. group2 45
S30 Schemes - Effect size 48
S31 Brief descriptions of the network metrics examined 51
S32 Metrics - Number of ICCs > 0.8 52
S33 Metrics - Number of ICCs > 0.6 53
S34 Metrics - ICC Mean 54
S35 Metrics - ICC Median 54
S36 Metrics - ICC group1 vs. group2 55
S37 Metrics - Effect size 57
S38 Metrics - Number of ICCs > 0.8 59
S39 Metrics - Number of ICCs > 0.6 60
S40 Metrics - ICC Mean 63
S41 Metrics - ICC Median 64

1 Parcellations - Node Definition

1.1 ICC Density distribution

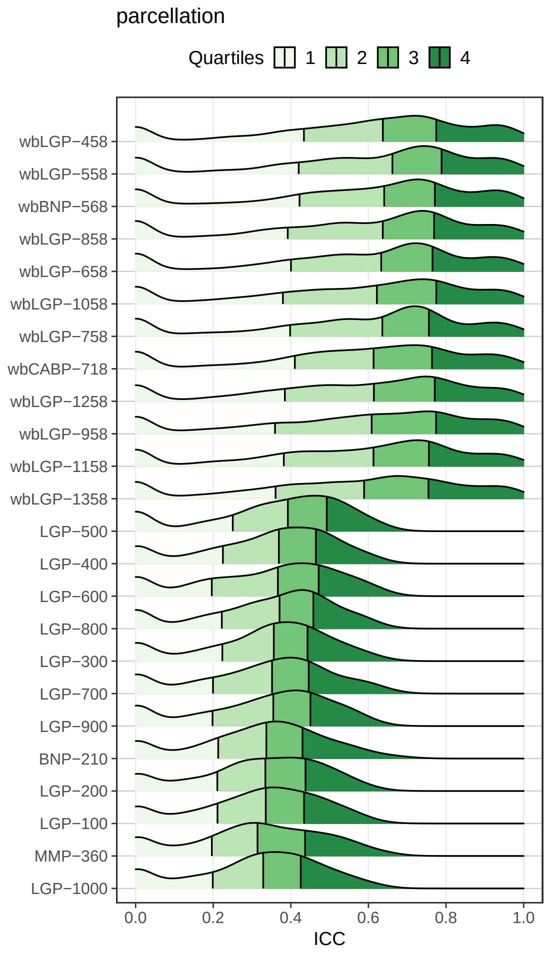

Fig. S1. Parcellation - Density distribution

1.2 ICC Almost Perfect (ICCs > 0.8)

Table S1. Parcellation - Number of ICCs > 0.8

parcellation	n	ratio
wbLGP-558	540	0.2295918
wbLGP-458	519	0.2206633
wbBNP-568	511	0.2172619
wbLGP-958	486	0.2066327
wbLGP-1058	484	0.2057823
wbLGP-1258	482	0.2049320
wbLGP-858	478	0.2032313
wbCABP-718	463	0.1968537
wbLGP-658	444	0.1887755
wbLGP-1358	440	0.1870748
wbLGP-1158	416	0.1768707
wbLGP-758	413	0.1755952

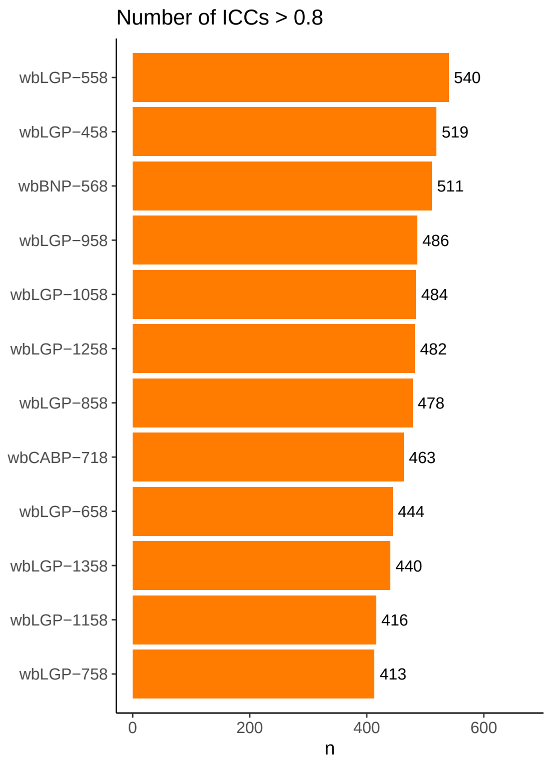

Fig. S2. Parcellation - Number of ICCs > 0.8

1.3 Substantial or Above (ICCs > 0.6)

Table S2. Parcellation - Number of ICCs > 0.6

parcellation	n	ratio
wbLGP-458	1328	0.5646259
wbLGP-558	1320	0.5612245
wbBNP-568	1298	0.5518707
wbLGP-858	1263	0.5369898
wbLGP-758	1248	0.5306122
wbLGP-658	1245	0.5293367
wbLGP-1058	1239	0.5267857
wbCABP-718	1224	0.5204082
wbLGP-1258	1216	0.5170068
wbLGP-1158	1211	0.5148810
wbLGP-958	1209	0.5140306
wbLGP-1358	1141	0.4851190
LGP-500	97	0.0412415
LGP-700	92	0.0391156
LGP-600	85	0.0361395
BNP-210	71	0.0301871
MMP-360	66	0.0280612
LGP-400	51	0.0216837
LGP-300	47	0.0199830
LGP-800	46	0.0195578
LGP-900	38	0.0161565
LGP-1000	37	0.0157313
LGP-200	24	0.0102041
LGP-100	21	0.0089286

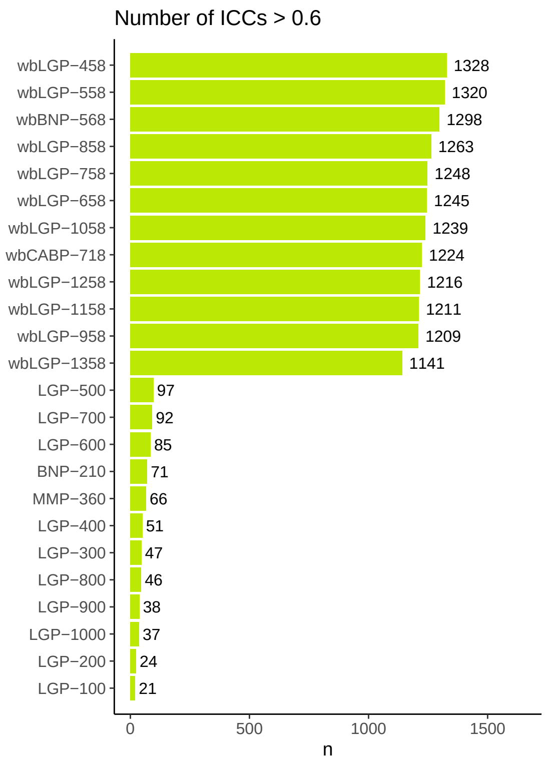

Fig. S3. Parcellation - Number of ICCs > 0.6

1.4 Descriptive statistics Mean

Table S3. Parcellation - ICC Mean

parcellation	variable	n	mean	mean_z2r
wbLGP-458	ICC.z	2352	0.813	0.6712419
wbLGP-558	ICC.z	2352	0.812	0.6706921
wbBNP-568	ICC.z	2352	0.800	0.6640368
wbLGP-858	ICC.z	2352	0.777	0.6509814
wbLGP-658	ICC.z	2352	0.775	0.6498275
wbLGP-1058	ICC.z	2352	0.770	0.6469295
wbCABP-718	ICC.z	2352	0.765	0.6440126
wbLGP-758	ICC.z	2352	0.765	0.6440126
wbLGP-1258	ICC.z	2352	0.764	0.6434270
wbLGP-958	ICC.z	2352	0.760	0.6410770
wbLGP-1158	ICC.z	2352	0.746	0.6327565
wbLGP-1358	ICC.z	2352	0.731	0.6236768
LGP-500	ICC.z	2352	0.379	0.3618387
LGP-400	ICC.z	2352	0.356	0.3416859
LGP-600	ICC.z	2352	0.354	0.3399182
LGP-800	ICC.z	2352	0.354	0.3399182
LGP-300	ICC.z	2352	0.347	0.3337123
LGP-700	ICC.z	2352	0.344	0.3310437
LGP-900	ICC.z	2352	0.340	0.3274774
BNP-210	ICC.z	2352	0.335	0.3230063
LGP-200	ICC.z	2352	0.330	0.3185208
LGP-100	ICC.z	2352	0.329	0.3176219
MMP-360	ICC.z	2352	0.326	0.3149220
LGP-1000	ICC.z	2352	0.324	0.3131193

1.5 Descriptive statistics Median

Table S4. Parcellation - ICC Median

parcellation	variable	n	median
wbLGP-558	ICC	2352	0.662
wbBNP-568	ICC	2352	0.641
wbLGP-458	ICC	2352	0.637
wbLGP-858	ICC	2352	0.637
wbLGP-758	ICC	2352	0.636
wbLGP-658	ICC	2352	0.633
wbLGP-1058	ICC	2352	0.622
wbLGP-1258	ICC	2352	0.614
wbCABP-718	ICC	2352	0.613
wbLGP-1158	ICC	2352	0.613
wbLGP-958	ICC	2352	0.608
wbLGP-1358	ICC	2352	0.589
LGP-500	ICC	2352	0.392
LGP-800	ICC	2352	0.371
LGP-400	ICC	2352	0.369
LGP-600	ICC	2352	0.367
LGP-300	ICC	2352	0.356
LGP-900	ICC	2352	0.355
LGP-700	ICC	2352	0.352
BNP-210	ICC	2352	0.337
LGP-100	ICC	2352	0.335
LGP-200	ICC	2352	0.334
LGP-1000	ICC	2352	0.329
MMP-360	ICC	2352	0.314

1.6 Friedman Test

Table S5. Parcellation - Friedman Test

.y.	n	statistic	df	p	method
ICC.z	2352	20379.07	23	0	Friedman test

1.7 Friedman Test Effect size

Table S6. Parcellation - Friedman Test Effect size

.y.	n	effsize	method	magnitude
ICC.z	2352	0.3767205	Kendall W	moderate

1.8 Paired Wilcoxon signed rank test

Table S7. Parcellation - Paired Wilcoxon signed rank test

group1	group2	n1	statistic	alternative	p	p.adj	p.adj.signif
BNP-210	LGP-100	2352	1302001	two.sided	1.60e-02	1.0000000	ns
BNP-210	LGP-1000	2352	1379617	two.sided	0.00e+00	0.0000030	****
BNP-210	LGP-200	2352	1330962	two.sided	2.00e-03	0.5520000	ns
BNP-210	LGP-300	2352	1047149	two.sided	0.00e+00	0.0000037	****
BNP-210	LGP-400	2352	928029	two.sided	0.00e+00	0.0000000	****
BNP-210	LGP-500	2352	686156	two.sided	0.00e+00	0.0000000	****
BNP-210	LGP-600	2352	1004541	two.sided	0.00e+00	0.0000000	****
BNP-210	LGP-700	2352	1105948	two.sided	1.76e-04	0.0485760	*
BNP-210	LGP-800	2352	945406	two.sided	0.00e+00	0.0000000	****
BNP-210	LGP-900	2352	1144573	two.sided	8.00e-03	1.0000000	ns
BNP-210	MMP-360	2352	1340035	two.sided	2.40e-04	0.0662400	ns
BNP-210	wbBNP-568	2352	160500	two.sided	0.00e+00	0.0000000	****
BNP-210	wbCABP-718	2352	162989	two.sided	0.00e+00	0.0000000	****
BNP-210	wbLGP-1058	2352	176518	two.sided	0.00e+00	0.0000000	****
BNP-210	wbLGP-1158	2352	199995	two.sided	0.00e+00	0.0000000	****
BNP-210	wbLGP-1258	2352	180282	two.sided	0.00e+00	0.0000000	****
BNP-210	wbLGP-1358	2352	232751	two.sided	0.00e+00	0.0000000	****
BNP-210	wbLGP-458	2352	145336	two.sided	0.00e+00	0.0000000	****
BNP-210	wbLGP-558	2352	148873	two.sided	0.00e+00	0.0000000	****
BNP-210	wbLGP-658	2352	175789	two.sided	0.00e+00	0.0000000	****
BNP-210	wbLGP-758	2352	180496	two.sided	0.00e+00	0.0000000	****
BNP-210	wbLGP-858	2352	170819	two.sided	0.00e+00	0.0000000	****
BNP-210	wbLGP-958	2352	189206	two.sided	0.00e+00	0.0000000	****
LGP-100	LGP-1000	2352	1315278	two.sided	6.00e-03	1.0000000	ns
LGP-100	LGP-200	2352	1255194	two.sided	5.26e-01	1.0000000	ns
LGP-100	LGP-300	2352	1010235	two.sided	0.00e+00	0.0000000	****
LGP-100	LGP-400	2352	912286	two.sided	0.00e+00	0.0000000	****
LGP-100	LGP-500	2352	659721	two.sided	0.00e+00	0.0000000	****
LGP-100	LGP-600	2352	981916	two.sided	0.00e+00	0.0000000	****
LGP-100	LGP-700	2352	1129951	two.sided	4.59e-04	0.1266840	ns
LGP-100	LGP-800	2352	994724	two.sided	0.00e+00	0.0000000	****
LGP-100	LGP-900	2352	1156218	two.sided	1.40e-02	1.0000000	ns
LGP-100	MMP-360	2352	1275769	two.sided	1.06e-01	1.0000000	ns
LGP-100	wbBNP-568	2352	159540	two.sided	0.00e+00	0.0000000	****
LGP-100	wbCABP-718	2352	161073	two.sided	0.00e+00	0.0000000	****
LGP-100	wbLGP-1058	2352	170565	two.sided	0.00e+00	0.0000000	****
LGP-100	wbLGP-1158	2352	187780	two.sided	0.00e+00	0.0000000	****
LGP-100	wbLGP-1258	2352	168428	two.sided	0.00e+00	0.0000000	****
LGP-100	wbLGP-1358	2352	215782	two.sided	0.00e+00	0.0000000	****
LGP-100	wbLGP-458	2352	130296	two.sided	0.00e+00	0.0000000	****

Table S7. Parcellation - Paired Wilcoxon signed rank test *(continued)*

group1	group2	n1	statistic	alternative	p	p.adj	p.adj.signif
LGP-100	wbLGP-558	2352	136606	two.sided	0.00e+00	0.0000000	****
LGP-100	wbLGP-658	2352	172094	two.sided	0.00e+00	0.0000000	****
LGP-100	wbLGP-758	2352	179105	two.sided	0.00e+00	0.0000000	****
LGP-100	wbLGP-858	2352	167470	two.sided	0.00e+00	0.0000000	****
LGP-100	wbLGP-958	2352	185024	two.sided	0.00e+00	0.0000000	****
LGP-1000	LGP-200	2352	1144843	two.sided	5.00e-03	1.0000000	ns
LGP-1000	LGP-300	2352	907429	two.sided	0.00e+00	0.0000000	****
LGP-1000	LGP-400	2352	756937	two.sided	0.00e+00	0.0000000	****
LGP-1000	LGP-500	2352	564982	two.sided	0.00e+00	0.0000000	****
LGP-1000	LGP-600	2352	766240	two.sided	0.00e+00	0.0000000	****
LGP-1000	LGP-700	2352	813046	two.sided	0.00e+00	0.0000000	****
LGP-1000	LGP-800	2352	724898	two.sided	0.00e+00	0.0000000	****
LGP-1000	LGP-900	2352	862540	two.sided	0.00e+00	0.0000000	****
LGP-1000	MMP-360	2352	1194004	two.sided	6.30e-01	1.0000000	ns
LGP-1000	wbBNP-568	2352	146449	two.sided	0.00e+00	0.0000000	****
LGP-1000	wbCABP-718	2352	133629	two.sided	0.00e+00	0.0000000	****
LGP-1000	wbLGP-1058	2352	143488	two.sided	0.00e+00	0.0000000	****
LGP-1000	wbLGP-1158	2352	152381	two.sided	0.00e+00	0.0000000	****
LGP-1000	wbLGP-1258	2352	140457	two.sided	0.00e+00	0.0000000	****
LGP-1000	wbLGP-1358	2352	180243	two.sided	0.00e+00	0.0000000	****
LGP-1000	wbLGP-458	2352	126403	two.sided	0.00e+00	0.0000000	****
LGP-1000	wbLGP-558	2352	130477	two.sided	0.00e+00	0.0000000	****
LGP-1000	wbLGP-658	2352	146959	two.sided	0.00e+00	0.0000000	****
LGP-1000	wbLGP-758	2352	146560	two.sided	0.00e+00	0.0000000	****
LGP-1000	wbLGP-858	2352	144497	two.sided	0.00e+00	0.0000000	****
LGP-1000	wbLGP-958	2352	151257	two.sided	0.00e+00	0.0000000	****
LGP-200	LGP-300	2352	925471	two.sided	0.00e+00	0.0000000	****
LGP-200	LGP-400	2352	832641	two.sided	0.00e+00	0.0000000	****
LGP-200	LGP-500	2352	576900	two.sided	0.00e+00	0.0000000	****
LGP-200	LGP-600	2352	932917	two.sided	0.00e+00	0.0000000	****
LGP-200	LGP-700	2352	1091274	two.sided	3.40e-06	0.0009301	***
LGP-200	LGP-800	2352	917094	two.sided	0.00e+00	0.0000000	****
LGP-200	LGP-900	2352	1112972	two.sided	1.30e-04	0.0358800	*
LGP-200	MMP-360	2352	1268709	two.sided	2.33e-01	1.0000000	ns
LGP-200	wbBNP-568	2352	144847	two.sided	0.00e+00	0.0000000	****
LGP-200	wbCABP-718	2352	149133	two.sided	0.00e+00	0.0000000	****
LGP-200	wbLGP-1058	2352	160610	two.sided	0.00e+00	0.0000000	****
LGP-200	wbLGP-1158	2352	180943	two.sided	0.00e+00	0.0000000	****
LGP-200	wbLGP-1258	2352	164714	two.sided	0.00e+00	0.0000000	****
LGP-200	wbLGP-1358	2352	207338	two.sided	0.00e+00	0.0000000	****
LGP-200	wbLGP-458	2352	130665	two.sided	0.00e+00	0.0000000	****

Table S7. Parcellation - Paired Wilcoxon signed rank test *(continued)*

group1	group2	n1	statistic	alternative	p	p.adj	p.adj.signif
LGP-200	wbLGP-558	2352	131597	two.sided	0.00e+00	0.0000000	****
LGP-200	wbLGP-658	2352	154264	two.sided	0.00e+00	0.0000000	****
LGP-200	wbLGP-758	2352	164041	two.sided	0.00e+00	0.0000000	****
LGP-200	wbLGP-858	2352	159660	two.sided	0.00e+00	0.0000000	****
LGP-200	wbLGP-958	2352	176411	two.sided	0.00e+00	0.0000000	****
LGP-300	LGP-400	2352	999180	two.sided	0.00e+00	0.0000000	****
LGP-300	LGP-500	2352	629869	two.sided	0.00e+00	0.0000000	****
LGP-300	LGP-600	2352	1060070	two.sided	2.00e-07	0.0000602	****
LGP-300	LGP-700	2352	1259128	two.sided	1.03e-01	1.0000000	ns
LGP-300	LGP-800	2352	1069448	two.sided	2.00e-07	0.0000538	****
LGP-300	LGP-900	2352	1299549	two.sided	6.00e-03	1.0000000	ns
LGP-300	MMP-360	2352	1483967	two.sided	0.00e+00	0.0000000	****
LGP-300	wbBNP-568	2352	164589	two.sided	0.00e+00	0.0000000	****
LGP-300	wbCABP-718	2352	158079	two.sided	0.00e+00	0.0000000	****
LGP-300	wbLGP-1058	2352	172650	two.sided	0.00e+00	0.0000000	****
LGP-300	wbLGP-1158	2352	196899	two.sided	0.00e+00	0.0000000	****
LGP-300	wbLGP-1258	2352	176738	two.sided	0.00e+00	0.0000000	****
LGP-300	wbLGP-1358	2352	227257	two.sided	0.00e+00	0.0000000	****
LGP-300	wbLGP-458	2352	143518	two.sided	0.00e+00	0.0000000	****
LGP-300	wbLGP-558	2352	144041	two.sided	0.00e+00	0.0000000	****
LGP-300	wbLGP-658	2352	173281	two.sided	0.00e+00	0.0000000	****
LGP-300	wbLGP-758	2352	170528	two.sided	0.00e+00	0.0000000	****
LGP-300	wbLGP-858	2352	163732	two.sided	0.00e+00	0.0000000	****
LGP-300	wbLGP-958	2352	186563	two.sided	0.00e+00	0.0000000	****
LGP-400	LGP-500	2352	767221	two.sided	0.00e+00	0.0000000	****
LGP-400	LGP-600	2352	1251633	two.sided	2.34e-01	1.0000000	ns
LGP-400	LGP-700	2352	1517947	two.sided	0.00e+00	0.0000000	****
LGP-400	LGP-800	2352	1303871	two.sided	1.20e-02	1.0000000	ns
LGP-400	LGP-900	2352	1477786	two.sided	0.00e+00	0.0000000	****
LGP-400	MMP-360	2352	1583190	two.sided	0.00e+00	0.0000000	****
LGP-400	wbBNP-568	2352	178030	two.sided	0.00e+00	0.0000000	****
LGP-400	wbCABP-718	2352	168997	two.sided	0.00e+00	0.0000000	****
LGP-400	wbLGP-1058	2352	190089	two.sided	0.00e+00	0.0000000	****
LGP-400	wbLGP-1158	2352	212433	two.sided	0.00e+00	0.0000000	****
LGP-400	wbLGP-1258	2352	188919	two.sided	0.00e+00	0.0000000	****
LGP-400	wbLGP-1358	2352	246801	two.sided	0.00e+00	0.0000000	****
LGP-400	wbLGP-458	2352	151198	two.sided	0.00e+00	0.0000000	****
LGP-400	wbLGP-558	2352	159132	two.sided	0.00e+00	0.0000000	****
LGP-400	wbLGP-658	2352	190283	two.sided	0.00e+00	0.0000000	****
LGP-400	wbLGP-758	2352	185190	two.sided	0.00e+00	0.0000000	****
LGP-400	wbLGP-858	2352	182103	two.sided	0.00e+00	0.0000000	****
LGP-400	wbLGP-958	2352	201977	two.sided	0.00e+00	0.0000000	****

Table S7. Parcellation - Paired Wilcoxon signed rank test *(continued)*

group1	group2	n1	statistic	alternative	p	p.adj	p.adj.signif
LGP-500	LGP-600	2352	1636990	two.sided	0.00e+00	0.0000000	****
LGP-500	LGP-700	2352	1775407	two.sided	0.00e+00	0.0000000	****
LGP-500	LGP-800	2352	1732442	two.sided	0.00e+00	0.0000000	****
LGP-500	LGP-900	2352	1731306	two.sided	0.00e+00	0.0000000	****
LGP-500	MMP-360	2352	1758329	two.sided	0.00e+00	0.0000000	****
LGP-500	wbBNP-568	2352	195928	two.sided	0.00e+00	0.0000000	****
LGP-500	wbCABP-718	2352	187217	two.sided	0.00e+00	0.0000000	****
LGP-500	wbLGP-1058	2352	202651	two.sided	0.00e+00	0.0000000	****
LGP-500	wbLGP-1158	2352	224307	two.sided	0.00e+00	0.0000000	****
LGP-500	wbLGP-1258	2352	206925	two.sided	0.00e+00	0.0000000	****
LGP-500	wbLGP-1358	2352	273700	two.sided	0.00e+00	0.0000000	****
LGP-500	wbLGP-458	2352	179170	two.sided	0.00e+00	0.0000000	****
LGP-500	wbLGP-558	2352	167854	two.sided	0.00e+00	0.0000000	****
LGP-500	wbLGP-658	2352	207577	two.sided	0.00e+00	0.0000000	****
LGP-500	wbLGP-758	2352	205183	two.sided	0.00e+00	0.0000000	****
LGP-500	wbLGP-858	2352	190097	two.sided	0.00e+00	0.0000000	****
LGP-500	wbLGP-958	2352	223872	two.sided	0.00e+00	0.0000000	****
LGP-600	LGP-700	2352	1517047	two.sided	0.00e+00	0.0000000	****
LGP-600	LGP-800	2352	1268374	two.sided	2.70e-02	1.0000000	ns
LGP-600	LGP-900	2352	1454702	two.sided	0.00e+00	0.0000000	****
LGP-600	MMP-360	2352	1486050	two.sided	0.00e+00	0.0000000	****
LGP-600	wbBNP-568	2352	151395	two.sided	0.00e+00	0.0000000	****
LGP-600	wbCABP-718	2352	146032	two.sided	0.00e+00	0.0000000	****
LGP-600	wbLGP-1058	2352	151532	two.sided	0.00e+00	0.0000000	****
LGP-600	wbLGP-1158	2352	174886	two.sided	0.00e+00	0.0000000	****
LGP-600	wbLGP-1258	2352	164221	two.sided	0.00e+00	0.0000000	****
LGP-600	wbLGP-1358	2352	210481	two.sided	0.00e+00	0.0000000	****
LGP-600	wbLGP-458	2352	142445	two.sided	0.00e+00	0.0000000	****
LGP-600	wbLGP-558	2352	138450	two.sided	0.00e+00	0.0000000	****
LGP-600	wbLGP-658	2352	156775	two.sided	0.00e+00	0.0000000	****
LGP-600	wbLGP-758	2352	158441	two.sided	0.00e+00	0.0000000	****
LGP-600	wbLGP-858	2352	148288	two.sided	0.00e+00	0.0000000	****
LGP-600	wbLGP-958	2352	145886	two.sided	0.00e+00	0.0000000	****
LGP-700	LGP-800	2352	994539	two.sided	0.00e+00	0.0000000	****
LGP-700	LGP-900	2352	1232646	two.sided	3.15e-01	1.0000000	ns
LGP-700	MMP-360	2352	1426998	two.sided	0.00e+00	0.0000000	****
LGP-700	wbBNP-568	2352	161398	two.sided	0.00e+00	0.0000000	****
LGP-700	wbCABP-718	2352	152205	two.sided	0.00e+00	0.0000000	****
LGP-700	wbLGP-1058	2352	163976	two.sided	0.00e+00	0.0000000	****
LGP-700	wbLGP-1158	2352	185951	two.sided	0.00e+00	0.0000000	****
LGP-700	wbLGP-1258	2352	167287	two.sided	0.00e+00	0.0000000	****
LGP-700	wbLGP-1358	2352	219346	two.sided	0.00e+00	0.0000000	****

Table S7. Parcellation - Paired Wilcoxon signed rank test *(continued)*

group1	group2	n1	statistic	alternative	p	p.adj	p.adj.signif
LGP-700	wbLGP-458	2352	142001	two.sided	0.00e+00	0.0000000	****
LGP-700	wbLGP-558	2352	145607	two.sided	0.00e+00	0.0000000	****
LGP-700	wbLGP-658	2352	169299	two.sided	0.00e+00	0.0000000	****
LGP-700	wbLGP-758	2352	169104	two.sided	0.00e+00	0.0000000	****
LGP-700	wbLGP-858	2352	164010	two.sided	0.00e+00	0.0000000	****
LGP-700	wbLGP-958	2352	173569	two.sided	0.00e+00	0.0000000	****
LGP-800	LGP-900	2352	1418708	two.sided	0.00e+00	0.0000000	****
LGP-800	MMP-360	2352	1553788	two.sided	0.00e+00	0.0000000	****
LGP-800	wbBNP-568	2352	181300	two.sided	0.00e+00	0.0000000	****
LGP-800	wbCABP-718	2352	172356	two.sided	0.00e+00	0.0000000	****
LGP-800	wbLGP-1058	2352	188254	two.sided	0.00e+00	0.0000000	****
LGP-800	wbLGP-1158	2352	193741	two.sided	0.00e+00	0.0000000	****
LGP-800	wbLGP-1258	2352	188270	two.sided	0.00e+00	0.0000000	****
LGP-800	wbLGP-1358	2352	242146	two.sided	0.00e+00	0.0000000	****
LGP-800	wbLGP-458	2352	162709	two.sided	0.00e+00	0.0000000	****
LGP-800	wbLGP-558	2352	160927	two.sided	0.00e+00	0.0000000	****
LGP-800	wbLGP-658	2352	189416	two.sided	0.00e+00	0.0000000	****
LGP-800	wbLGP-758	2352	190433	two.sided	0.00e+00	0.0000000	****
LGP-800	wbLGP-858	2352	183382	two.sided	0.00e+00	0.0000000	****
LGP-800	wbLGP-958	2352	201777	two.sided	0.00e+00	0.0000000	****
LGP-900	MMP-360	2352	1411598	two.sided	0.00e+00	0.0000000	****
LGP-900	wbBNP-568	2352	161762	two.sided	0.00e+00	0.0000000	****
LGP-900	wbCABP-718	2352	150635	two.sided	0.00e+00	0.0000000	****
LGP-900	wbLGP-1058	2352	167674	two.sided	0.00e+00	0.0000000	****
LGP-900	wbLGP-1158	2352	175152	two.sided	0.00e+00	0.0000000	****
LGP-900	wbLGP-1258	2352	162633	two.sided	0.00e+00	0.0000000	****
LGP-900	wbLGP-1358	2352	210734	two.sided	0.00e+00	0.0000000	****
LGP-900	wbLGP-458	2352	143322	two.sided	0.00e+00	0.0000000	****
LGP-900	wbLGP-558	2352	145640	two.sided	0.00e+00	0.0000000	****
LGP-900	wbLGP-658	2352	166904	two.sided	0.00e+00	0.0000000	****
LGP-900	wbLGP-758	2352	172439	two.sided	0.00e+00	0.0000000	****
LGP-900	wbLGP-858	2352	161857	two.sided	0.00e+00	0.0000000	****
LGP-900	wbLGP-958	2352	177763	two.sided	0.00e+00	0.0000000	****
MMP-360	wbBNP-568	2352	153083	two.sided	0.00e+00	0.0000000	****
MMP-360	wbCABP-718	2352	137815	two.sided	0.00e+00	0.0000000	****
MMP-360	wbLGP-1058	2352	158332	two.sided	0.00e+00	0.0000000	****
MMP-360	wbLGP-1158	2352	185386	two.sided	0.00e+00	0.0000000	****
MMP-360	wbLGP-1258	2352	166968	two.sided	0.00e+00	0.0000000	****
MMP-360	wbLGP-1358	2352	219183	two.sided	0.00e+00	0.0000000	****
MMP-360	wbLGP-458	2352	137964	two.sided	0.00e+00	0.0000000	****
MMP-360	wbLGP-558	2352	138865	two.sided	0.00e+00	0.0000000	****

Table S7. Parcellation - Paired Wilcoxon signed rank test *(continued)*

group1	group2	n1	statistic	alternative	p	p.adj	p.adj.signif
MMP-360	wbLGP-658	2352	156009	two.sided	0.00e+00	0.0000000	****
MMP-360	wbLGP-758	2352	164978	two.sided	0.00e+00	0.0000000	****
MMP-360	wbLGP-858	2352	157870	two.sided	0.00e+00	0.0000000	****
MMP-360	wbLGP-958	2352	182605	two.sided	0.00e+00	0.0000000	****
wbBNP-568	wbCABP-718	2352	1639241	two.sided	0.00e+00	0.0000000	****
wbBNP-568	wbLGP-1058	2352	1547859	two.sided	0.00e+00	0.0000000	****
wbBNP-568	wbLGP-1158	2352	1533796	two.sided	0.00e+00	0.0000000	****
wbBNP-568	wbLGP-1258	2352	1447228	two.sided	0.00e+00	0.0000000	****
wbBNP-568	wbLGP-1358	2352	1650972	two.sided	0.00e+00	0.0000000	****
wbBNP-568	wbLGP-458	2352	1125170	two.sided	1.22e-05	0.0033672	**
wbBNP-568	wbLGP-558	2352	1098196	two.sided	2.40e-06	0.0006486	***
wbBNP-568	wbLGP-658	2352	1550059	two.sided	0.00e+00	0.0000000	****
wbBNP-568	wbLGP-758	2352	1595774	two.sided	0.00e+00	0.0000000	****
wbBNP-568	wbLGP-858	2352	1458694	two.sided	0.00e+00	0.0000000	****
wbBNP-568	wbLGP-958	2352	1661566	two.sided	0.00e+00	0.0000000	****
wbCABP-718	wbLGP-1058	2352	1166229	two.sided	8.90e-02	1.0000000	ns
wbCABP-718	wbLGP-1158	2352	1266087	two.sided	3.20e-01	1.0000000	ns
wbCABP-718	wbLGP-1258	2352	1176085	two.sided	5.60e-02	1.0000000	ns
wbCABP-718	wbLGP-1358	2352	1400531	two.sided	1.00e-07	0.0000293	****
wbCABP-718	wbLGP-458	2352	831718	two.sided	0.00e+00	0.0000000	****
wbCABP-718	wbLGP-558	2352	757451	two.sided	0.00e+00	0.0000000	****
wbCABP-718	wbLGP-658	2352	1045849	two.sided	0.00e+00	0.0000006	****
wbCABP-718	wbLGP-758	2352	1166760	two.sided	5.80e-02	1.0000000	ns
wbCABP-718	wbLGP-858	2352	1068928	two.sided	4.00e-07	0.0001190	***
wbCABP-718	wbLGP-958	2352	1278722	two.sided	8.60e-02	1.0000000	ns
wbLGP-1058	wbLGP-1158	2352	1310431	two.sided	1.00e-02	1.0000000	ns
wbLGP-1058	wbLGP-1258	2352	1068697	two.sided	1.00e-07	0.0000157	****
wbLGP-1058	wbLGP-1358	2352	1503855	two.sided	0.00e+00	0.0000000	****
wbLGP-1058	wbLGP-458	2352	921542	two.sided	0.00e+00	0.0000000	****
wbLGP-1058	wbLGP-558	2352	830108	two.sided	0.00e+00	0.0000000	****
wbLGP-1058	wbLGP-658	2352	1070610	two.sided	3.00e-07	0.0000944	****
wbLGP-1058	wbLGP-758	2352	1177300	two.sided	2.97e-01	1.0000000	ns
wbLGP-1058	wbLGP-858	2352	975194	two.sided	0.00e+00	0.0000000	****
wbLGP-1058	wbLGP-958	2352	1341281	two.sided	5.59e-05	0.0154284	*
wbLGP-1158	wbLGP-1258	2352	1053122	two.sided	0.00e+00	0.0000005	****
wbLGP-1158	wbLGP-1358	2352	1451123	two.sided	0.00e+00	0.0000000	****
wbLGP-1158	wbLGP-458	2352	908290	two.sided	0.00e+00	0.0000000	****
wbLGP-1158	wbLGP-558	2352	892111	two.sided	0.00e+00	0.0000000	****
wbLGP-1158	wbLGP-658	2352	1078962	two.sided	3.00e-07	0.0000836	****
wbLGP-1158	wbLGP-758	2352	1178685	two.sided	1.16e-01	1.0000000	ns
wbLGP-1158	wbLGP-858	2352	1008073	two.sided	0.00e+00	0.0000000	****
wbLGP-1158	wbLGP-958	2352	1295606	two.sided	3.40e-02	1.0000000	ns

Table S7. Parcellation - Paired Wilcoxon signed rank test *(continued)*

group1	group2	n1	statistic	alternative	p	p.adj	p.adj.signif
wbLGP-1258	wbLGP-1358	2352	1612256	two.sided	0.00e+00	0.0000000	****
wbLGP-1258	wbLGP-458	2352	994969	two.sided	0.00e+00	0.0000000	****
wbLGP-1258	wbLGP-558	2352	957447	two.sided	0.00e+00	0.0000000	****
wbLGP-1258	wbLGP-658	2352	1172824	two.sided	4.40e-02	1.0000000	ns
wbLGP-1258	wbLGP-758	2352	1273752	two.sided	1.74e-01	1.0000000	ns
wbLGP-1258	wbLGP-858	2352	1150313	two.sided	8.00e-03	1.0000000	ns
wbLGP-1258	wbLGP-958	2352	1390511	two.sided	1.00e-07	0.0000197	****
wbLGP-1358	wbLGP-458	2352	824258	two.sided	0.00e+00	0.0000000	****
wbLGP-1358	wbLGP-558	2352	808992	two.sided	0.00e+00	0.0000000	****
wbLGP-1358	wbLGP-658	2352	968209	two.sided	0.00e+00	0.0000000	****
wbLGP-1358	wbLGP-758	2352	1031212	two.sided	0.00e+00	0.0000000	****
wbLGP-1358	wbLGP-858	2352	936260	two.sided	0.00e+00	0.0000000	****
wbLGP-1358	wbLGP-958	2352	1134304	two.sided	6.89e-04	0.1901640	ns
wbLGP-458	wbLGP-558	2352	1264741	two.sided	5.63e-01	1.0000000	ns
wbLGP-458	wbLGP-658	2352	1598752	two.sided	0.00e+00	0.0000000	****
wbLGP-458	wbLGP-758	2352	1629670	two.sided	0.00e+00	0.0000000	****
wbLGP-458	wbLGP-858	2352	1517308	two.sided	0.00e+00	0.0000000	****
wbLGP-458	wbLGP-958	2352	1684990	two.sided	0.00e+00	0.0000000	****
wbLGP-558	wbLGP-658	2352	1702013	two.sided	0.00e+00	0.0000000	****
wbLGP-558	wbLGP-758	2352	1693929	two.sided	0.00e+00	0.0000000	****
wbLGP-558	wbLGP-858	2352	1557712	two.sided	0.00e+00	0.0000000	****
wbLGP-558	wbLGP-958	2352	1716972	two.sided	0.00e+00	0.0000000	****
wbLGP-658	wbLGP-758	2352	1388101	two.sided	0.00e+00	0.0000049	****
wbLGP-658	wbLGP-858	2352	1241424	two.sided	5.82e-01	1.0000000	ns
wbLGP-658	wbLGP-958	2352	1515613	two.sided	0.00e+00	0.0000000	****
wbLGP-758	wbLGP-858	2352	970045	two.sided	0.00e+00	0.0000000	****
wbLGP-758	wbLGP-958	2352	1351987	two.sided	3.70e-06	0.0010295	**
wbLGP-858	wbLGP-958	2352	1494529	two.sided	0.00e+00	0.0000000	****

1.9 Kullback-leibler divergence Map

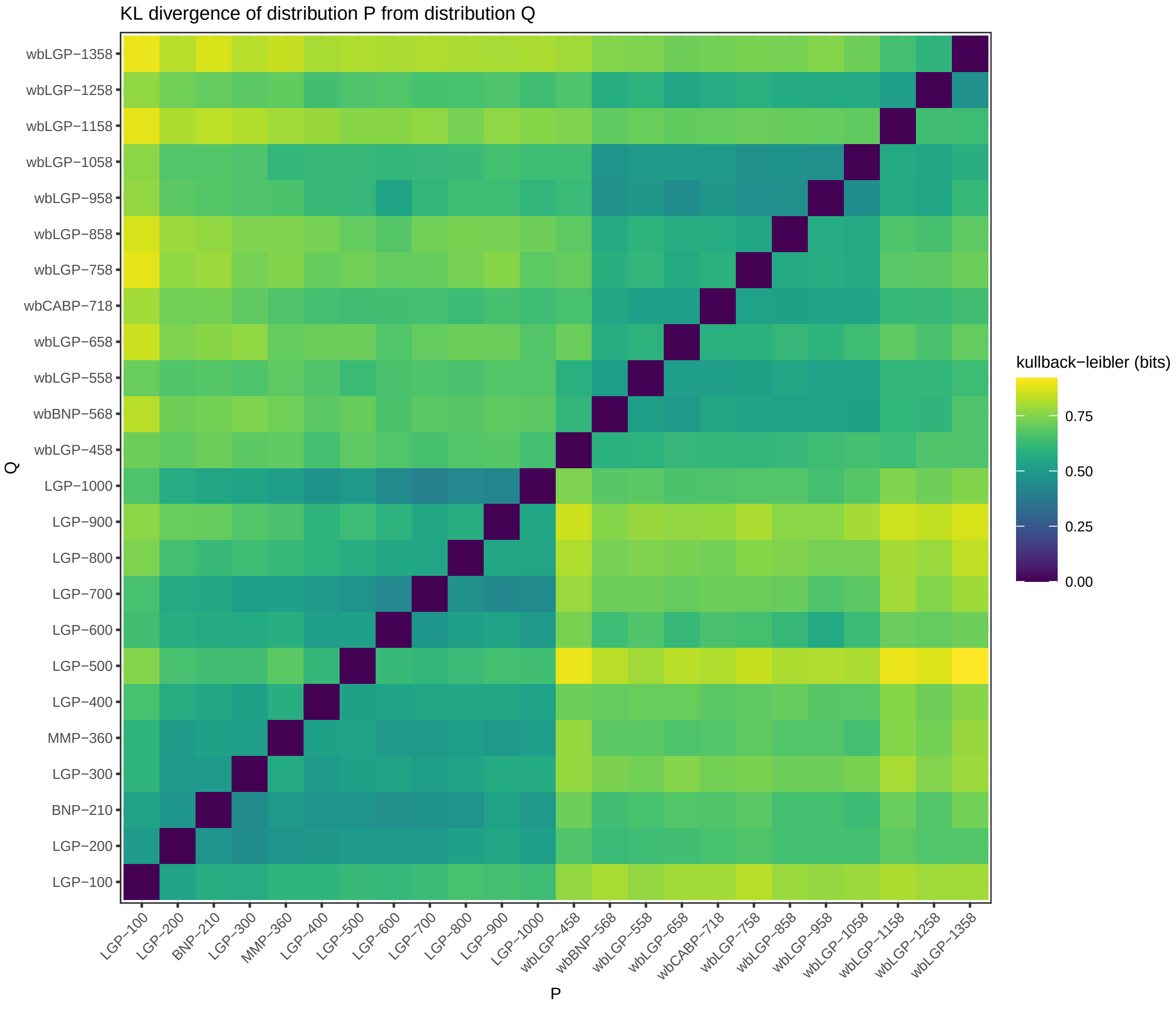

Fig. S4. Parcellation - KL divergence Map

1.10 Significance Map

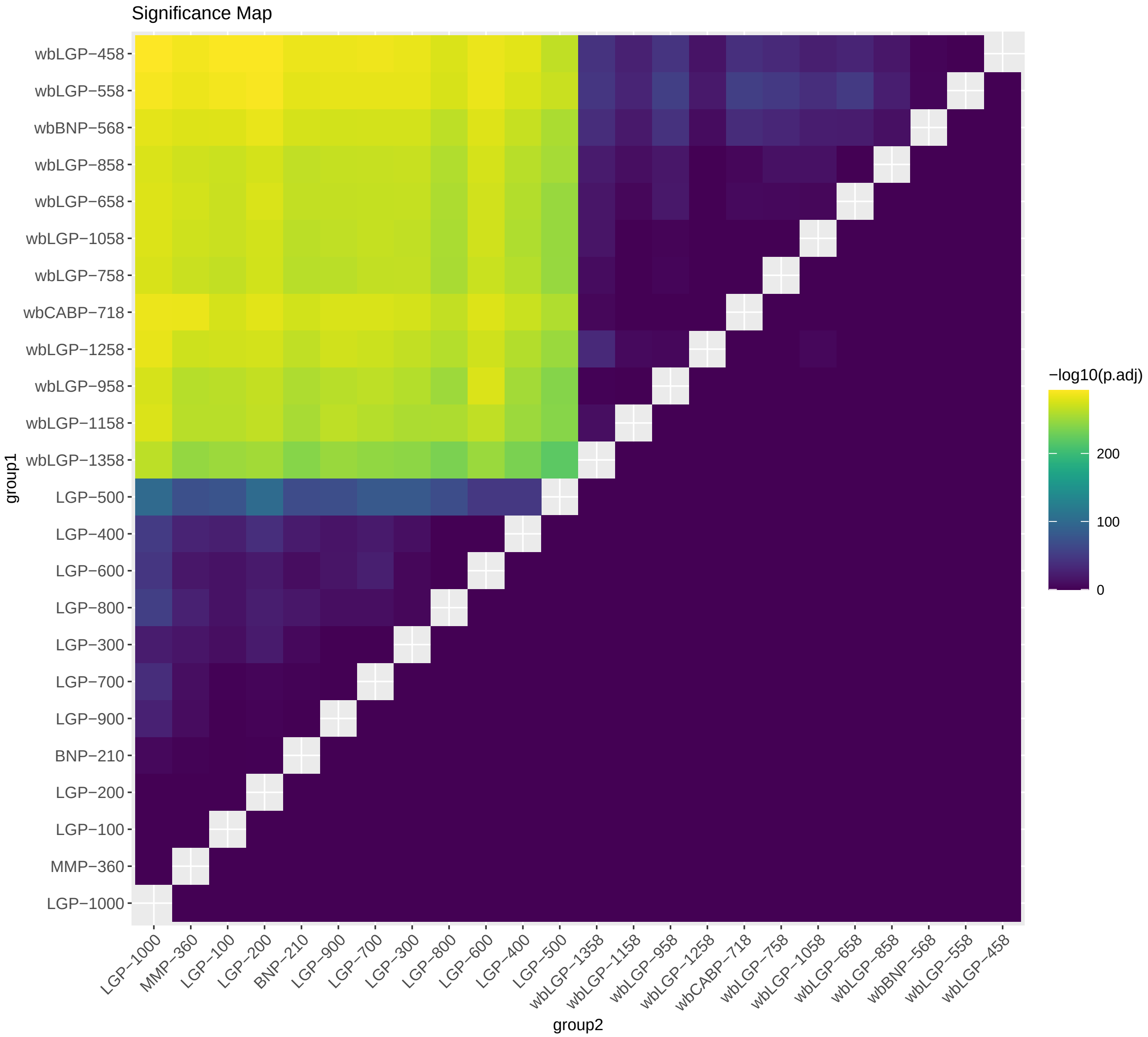

Fig. S5. Parcellation - Significance Map

1.11 Effect size

Table S8. Parcellation - Effect size

group1	group2	effsize	n1	n2	magnitude
BNP-210	LGP-100	0.0512884	2352	2352	small
BNP-210	LGP-1000	0.1207652	2352	2352	small
BNP-210	LGP-200	0.0626895	2352	2352	small
BNP-210	LGP-300	0.1141191	2352	2352	small
BNP-210	LGP-400	0.2082967	2352	2352	small
BNP-210	LGP-500	0.3653563	2352	2352	moderate
BNP-210	LGP-600	0.1408717	2352	2352	small

Table S8. Parcellation - Effect size *(continued)*

group1	group2	effsize	n1	n2	magnitude
BNP-210	LGP-700	0.0779897	2352	2352	small
BNP-210	LGP-800	0.1893357	2352	2352	small
BNP-210	LGP-900	0.0543280	2352	2352	small
BNP-210	MMP-360	0.0731563	2352	2352	small
BNP-210	wbBNP-568	0.7389896	2352	2352	large
BNP-210	wbCABP-718	0.7379236	2352	2352	large
BNP-210	wbLGP-1058	0.7216681	2352	2352	large
BNP-210	wbLGP-1158	0.7104950	2352	2352	large
BNP-210	wbLGP-1258	0.7231289	2352	2352	large
BNP-210	wbLGP-1358	0.6888160	2352	2352	large
BNP-210	wbLGP-458	0.7536559	2352	2352	large
BNP-210	wbLGP-558	0.7475750	2352	2352	large
BNP-210	wbLGP-658	0.7266246	2352	2352	large
BNP-210	wbLGP-758	0.7214303	2352	2352	large
BNP-210	wbLGP-858	0.7258208	2352	2352	large
BNP-210	wbLGP-958	0.7141604	2352	2352	large
LGP-100	LGP-1000	0.0539583	2352	2352	small
LGP-100	LGP-200	0.0129528	2352	2352	small
LGP-100	LGP-300	0.1500861	2352	2352	small
LGP-100	LGP-400	0.2208211	2352	2352	small
LGP-100	LGP-500	0.3913895	2352	2352	moderate
LGP-100	LGP-600	0.1695900	2352	2352	small
LGP-100	LGP-700	0.0698175	2352	2352	small
LGP-100	LGP-800	0.1689070	2352	2352	small
LGP-100	LGP-900	0.0495689	2352	2352	small
LGP-100	MMP-360	0.0317933	2352	2352	small
LGP-100	wbBNP-568	0.7436624	2352	2352	large
LGP-100	wbCABP-718	0.7394118	2352	2352	large
LGP-100	wbLGP-1058	0.7296547	2352	2352	large
LGP-100	wbLGP-1158	0.7220587	2352	2352	large
LGP-100	wbLGP-1258	0.7355677	2352	2352	large
LGP-100	wbLGP-1358	0.7044764	2352	2352	large
LGP-100	wbLGP-458	0.7640762	2352	2352	large
LGP-100	wbLGP-558	0.7569985	2352	2352	large
LGP-100	wbLGP-658	0.7310191	2352	2352	large
LGP-100	wbLGP-758	0.7275245	2352	2352	large
LGP-100	wbLGP-858	0.7326421	2352	2352	large
LGP-100	wbLGP-958	0.7209303	2352	2352	large
LGP-1000	LGP-200	0.0578365	2352	2352	small
LGP-1000	LGP-300	0.2105271	2352	2352	small
LGP-1000	LGP-400	0.3192842	2352	2352	moderate
LGP-1000	LGP-500	0.4487333	2352	2352	moderate
LGP-1000	LGP-600	0.3020832	2352	2352	moderate
LGP-1000	LGP-700	0.2719757	2352	2352	small
LGP-1000	LGP-800	0.3321805	2352	2352	moderate
LGP-1000	LGP-900	0.2340567	2352	2352	small
LGP-1000	MMP-360	0.0081145	2352	2352	small

Table S8. Parcellation - Effect size *(continued)*

group1	group2	effsize	n1	n2	magnitude
LGP-1000	wbBNP-568	0.7500285	2352	2352	large
LGP-1000	wbCABP-718	0.7568211	2352	2352	large
LGP-1000	wbLGP-1058	0.7435657	2352	2352	large
LGP-1000	wbLGP-1158	0.7443931	2352	2352	large
LGP-1000	wbLGP-1258	0.7509503	2352	2352	large
LGP-1000	wbLGP-1358	0.7255476	2352	2352	large
LGP-1000	wbLGP-458	0.7662544	2352	2352	large
LGP-1000	wbLGP-558	0.7623347	2352	2352	large
LGP-1000	wbLGP-658	0.7456084	2352	2352	large
LGP-1000	wbLGP-758	0.7432770	2352	2352	large
LGP-1000	wbLGP-858	0.7434569	2352	2352	large
LGP-1000	wbLGP-958	0.7395782	2352	2352	large
LGP-200	LGP-300	0.2081011	2352	2352	small
LGP-200	LGP-400	0.2759045	2352	2352	small
LGP-200	LGP-500	0.4511007	2352	2352	moderate
LGP-200	LGP-600	0.1986975	2352	2352	small
LGP-200	LGP-700	0.0956037	2352	2352	small
LGP-200	LGP-800	0.2215336	2352	2352	small
LGP-200	LGP-900	0.0807512	2352	2352	small
LGP-200	MMP-360	0.0232331	2352	2352	small
LGP-200	wbBNP-568	0.7524502	2352	2352	large
LGP-200	wbCABP-718	0.7494454	2352	2352	large
LGP-200	wbLGP-1058	0.7351284	2352	2352	large
LGP-200	wbLGP-1158	0.7272050	2352	2352	large
LGP-200	wbLGP-1258	0.7374204	2352	2352	large
LGP-200	wbLGP-1358	0.7088809	2352	2352	large
LGP-200	wbLGP-458	0.7639591	2352	2352	large
LGP-200	wbLGP-558	0.7610277	2352	2352	large
LGP-200	wbLGP-658	0.7432334	2352	2352	large
LGP-200	wbLGP-758	0.7366071	2352	2352	large
LGP-200	wbLGP-858	0.7386798	2352	2352	large
LGP-200	wbLGP-958	0.7282388	2352	2352	large
LGP-300	LGP-400	0.1569344	2352	2352	small
LGP-300	LGP-500	0.4014572	2352	2352	moderate
LGP-300	LGP-600	0.1086461	2352	2352	small
LGP-300	LGP-700	0.0305094	2352	2352	small
LGP-300	LGP-800	0.1105415	2352	2352	small
LGP-300	LGP-900	0.0588944	2352	2352	small
LGP-300	MMP-360	0.1802777	2352	2352	small
LGP-300	wbBNP-568	0.7393016	2352	2352	large
LGP-300	wbCABP-718	0.7409097	2352	2352	large
LGP-300	wbLGP-1058	0.7254031	2352	2352	large
LGP-300	wbLGP-1158	0.7146360	2352	2352	large
LGP-300	wbLGP-1258	0.7276375	2352	2352	large
LGP-300	wbLGP-1358	0.6950338	2352	2352	large
LGP-300	wbLGP-458	0.7545390	2352	2352	large

Table S8. Parcellation - Effect size *(continued)*

group1	group2	effsize	n1	n2	magnitude
LGP-300	wbLGP-558	0.7518696	2352	2352	large
LGP-300	wbLGP-658	0.7305186	2352	2352	large
LGP-300	wbLGP-758	0.7288353	2352	2352	large
LGP-300	wbLGP-858	0.7315947	2352	2352	large
LGP-300	wbLGP-958	0.7184744	2352	2352	large
LGP-400	LGP-500	0.3106815	2352	2352	moderate
LGP-400	LGP-600	0.0239758	2352	2352	small
LGP-400	LGP-700	0.2024827	2352	2352	small
LGP-400	LGP-800	0.0547714	2352	2352	small
LGP-400	LGP-900	0.1808123	2352	2352	small
LGP-400	MMP-360	0.2340978	2352	2352	small
LGP-400	wbBNP-568	0.7295883	2352	2352	large
LGP-400	wbCABP-718	0.7324939	2352	2352	large
LGP-400	wbLGP-1058	0.7127931	2352	2352	large
LGP-400	wbLGP-1158	0.7034513	2352	2352	large
LGP-400	wbLGP-1258	0.7163478	2352	2352	large
LGP-400	wbLGP-1358	0.6809360	2352	2352	large
LGP-400	wbLGP-458	0.7489080	2352	2352	large
LGP-400	wbLGP-558	0.7418199	2352	2352	large
LGP-400	wbLGP-658	0.7177687	2352	2352	large
LGP-400	wbLGP-758	0.7184835	2352	2352	large
LGP-400	wbLGP-858	0.7209195	2352	2352	large
LGP-400	wbLGP-958	0.7072074	2352	2352	large
LGP-500	LGP-600	0.3060236	2352	2352	moderate
LGP-500	LGP-700	0.4001344	2352	2352	moderate
LGP-500	LGP-800	0.3747741	2352	2352	moderate
LGP-500	LGP-900	0.3739231	2352	2352	moderate
LGP-500	MMP-360	0.3762905	2352	2352	moderate
LGP-500	wbBNP-568	0.7165713	2352	2352	large
LGP-500	wbCABP-718	0.7182115	2352	2352	large
LGP-500	wbLGP-1058	0.7002399	2352	2352	large
LGP-500	wbLGP-1158	0.6914885	2352	2352	large
LGP-500	wbLGP-1258	0.7026320	2352	2352	large
LGP-500	wbLGP-1358	0.6590763	2352	2352	large
LGP-500	wbLGP-458	0.7298496	2352	2352	large
LGP-500	wbLGP-558	0.7343705	2352	2352	large
LGP-500	wbLGP-658	0.7035363	2352	2352	large
LGP-500	wbLGP-758	0.7031792	2352	2352	large
LGP-500	wbLGP-858	0.7123423	2352	2352	large
LGP-500	wbLGP-958	0.6868862	2352	2352	large
LGP-600	LGP-700	0.2268428	2352	2352	small
LGP-600	LGP-800	0.0462445	2352	2352	small
LGP-600	LGP-900	0.1816469	2352	2352	small
LGP-600	MMP-360	0.1886473	2352	2352	small
LGP-600	wbBNP-568	0.7481082	2352	2352	large
LGP-600	wbCABP-718	0.7481815	2352	2352	large

Table S8. Parcellation - Effect size *(continued)*

group1	group2	effsize	n1	n2	magnitude
LGP-600	wbLGP-1058	0.7383695	2352	2352	large
LGP-600	wbLGP-1158	0.7294442	2352	2352	large
LGP-600	wbLGP-1258	0.7369489	2352	2352	large
LGP-600	wbLGP-1358	0.7042384	2352	2352	large
LGP-600	wbLGP-458	0.7557913	2352	2352	large
LGP-600	wbLGP-558	0.7562789	2352	2352	large
LGP-600	wbLGP-658	0.7398462	2352	2352	large
LGP-600	wbLGP-758	0.7355434	2352	2352	large
LGP-600	wbLGP-858	0.7421715	2352	2352	large
LGP-600	wbLGP-958	0.7452103	2352	2352	large
LGP-700	LGP-800	0.1482687	2352	2352	small
LGP-700	LGP-900	0.0223819	2352	2352	small
LGP-700	MMP-360	0.1459144	2352	2352	small
LGP-700	wbBNP-568	0.7385190	2352	2352	large
LGP-700	wbCABP-718	0.7428081	2352	2352	large
LGP-700	wbLGP-1058	0.7280828	2352	2352	large
LGP-700	wbLGP-1158	0.7198417	2352	2352	large
LGP-700	wbLGP-1258	0.7322622	2352	2352	large
LGP-700	wbLGP-1358	0.6979349	2352	2352	large
LGP-700	wbLGP-458	0.7555548	2352	2352	large
LGP-700	wbLGP-558	0.7506720	2352	2352	large
LGP-700	wbLGP-658	0.7296047	2352	2352	large
LGP-700	wbLGP-758	0.7284709	2352	2352	large
LGP-700	wbLGP-858	0.7297612	2352	2352	large
LGP-700	wbLGP-958	0.7241542	2352	2352	large
LGP-800	LGP-900	0.1523587	2352	2352	small
LGP-800	MMP-360	0.2294773	2352	2352	small
LGP-800	wbBNP-568	0.7247815	2352	2352	large
LGP-800	wbCABP-718	0.7278916	2352	2352	large
LGP-800	wbLGP-1058	0.7110976	2352	2352	large
LGP-800	wbLGP-1158	0.7148132	2352	2352	large
LGP-800	wbLGP-1258	0.7177082	2352	2352	large
LGP-800	wbLGP-1358	0.6815238	2352	2352	large
LGP-800	wbLGP-458	0.7424878	2352	2352	large
LGP-800	wbLGP-558	0.7403174	2352	2352	large
LGP-800	wbLGP-658	0.7160305	2352	2352	large
LGP-800	wbLGP-758	0.7131063	2352	2352	large
LGP-800	wbLGP-858	0.7184349	2352	2352	large
LGP-800	wbLGP-958	0.7032708	2352	2352	large
LGP-900	MMP-360	0.1404051	2352	2352	small
LGP-900	wbBNP-568	0.7387104	2352	2352	large
LGP-900	wbCABP-718	0.7433471	2352	2352	large
LGP-900	wbLGP-1058	0.7243857	2352	2352	large
LGP-900	wbLGP-1158	0.7255897	2352	2352	large
LGP-900	wbLGP-1258	0.7343608	2352	2352	large
LGP-900	wbLGP-1358	0.7026858	2352	2352	large
LGP-900	wbLGP-458	0.7545988	2352	2352	large

Table S8. Parcellation - Effect size *(continued)*

group1	group2	effsize	n1	n2	magnitude
LGP-900	wbLGP-558	0.7503933	2352	2352	large
LGP-900	wbLGP-658	0.7302955	2352	2352	large
LGP-900	wbLGP-758	0.7241196	2352	2352	large
LGP-900	wbLGP-858	0.7301608	2352	2352	large
LGP-900	wbLGP-958	0.7201412	2352	2352	large
MMP-360	wbBNP-568	0.7444282	2352	2352	large
MMP-360	wbCABP-718	0.7535082	2352	2352	large
MMP-360	wbLGP-1058	0.7311101	2352	2352	large
MMP-360	wbLGP-1158	0.7201675	2352	2352	large
MMP-360	wbLGP-1258	0.7319867	2352	2352	large
MMP-360	wbLGP-1358	0.6975871	2352	2352	large
MMP-360	wbLGP-458	0.7584484	2352	2352	large
MMP-360	wbLGP-558	0.7548697	2352	2352	large
MMP-360	wbLGP-658	0.7382470	2352	2352	large
MMP-360	wbLGP-758	0.7320983	2352	2352	large
MMP-360	wbLGP-858	0.7345617	2352	2352	large
MMP-360	wbLGP-958	0.7174438	2352	2352	large
wbBNP-568	wbCABP-718	0.2728574	2352	2352	small
wbBNP-568	wbLGP-1058	0.2162613	2352	2352	small
wbBNP-568	wbLGP-1158	0.1995484	2352	2352	small
wbBNP-568	wbLGP-1258	0.1386707	2352	2352	small
wbBNP-568	wbLGP-1358	0.2717290	2352	2352	small
wbBNP-568	wbLGP-458	0.0877119	2352	2352	small
wbBNP-568	wbLGP-558	0.0947171	2352	2352	small
wbBNP-568	wbLGP-658	0.2160494	2352	2352	small
wbBNP-568	wbLGP-758	0.2540569	2352	2352	small
wbBNP-568	wbLGP-858	0.1592633	2352	2352	small
wbBNP-568	wbLGP-958	0.2937065	2352	2352	small
wbCABP-718	wbLGP-1058	0.0359091	2352	2352	small
wbCABP-718	wbLGP-1158	0.0233259	2352	2352	small
wbCABP-718	wbLGP-1258	0.0388589	2352	2352	small
wbCABP-718	wbLGP-1358	0.1106258	2352	2352	small
wbCABP-718	wbLGP-458	0.2848362	2352	2352	small
wbCABP-718	wbLGP-558	0.3284945	2352	2352	moderate
wbCABP-718	wbLGP-658	0.1230697	2352	2352	small
wbCABP-718	wbLGP-758	0.0370190	2352	2352	small
wbCABP-718	wbLGP-858	0.0991309	2352	2352	small
wbCABP-718	wbLGP-958	0.0362152	2352	2352	small
wbLGP-1058	wbLGP-1158	0.0561301	2352	2352	small
wbLGP-1058	wbLGP-1258	0.1116554	2352	2352	small
wbLGP-1058	wbLGP-1358	0.1822538	2352	2352	small
wbLGP-1058	wbLGP-458	0.2180510	2352	2352	small
wbLGP-1058	wbLGP-558	0.2778507	2352	2352	small
wbLGP-1058	wbLGP-658	0.1051919	2352	2352	small
wbLGP-1058	wbLGP-758	0.0228152	2352	2352	small
wbLGP-1058	wbLGP-858	0.1661968	2352	2352	small
wbLGP-1058	wbLGP-958	0.0818575	2352	2352	small

Table S8. Parcellation - Effect size *(continued)*

group1	group2	effsize	n1	n2	magnitude
wbLGP-1158	wbLGP-1258	0.1252645	2352	2352	small
wbLGP-1158	wbLGP-1358	0.1480026	2352	2352	small
wbLGP-1158	wbLGP-458	0.2274392	2352	2352	small
wbLGP-1158	wbLGP-558	0.2439547	2352	2352	small
wbLGP-1158	wbLGP-658	0.1045024	2352	2352	small
wbLGP-1158	wbLGP-758	0.0322159	2352	2352	small
wbLGP-1158	wbLGP-858	0.1551948	2352	2352	small
wbLGP-1158	wbLGP-958	0.0414800	2352	2352	small
wbLGP-1258	wbLGP-1358	0.2590570	2352	2352	small
wbLGP-1258	wbLGP-458	0.1735670	2352	2352	small
wbLGP-1258	wbLGP-558	0.1977710	2352	2352	small
wbLGP-1258	wbLGP-658	0.0419666	2352	2352	small
wbLGP-1258	wbLGP-758	0.0287042	2352	2352	small
wbLGP-1258	wbLGP-858	0.0559503	2352	2352	small
wbLGP-1258	wbLGP-958	0.1063742	2352	2352	small
wbLGP-1358	wbLGP-458	0.2918365	2352	2352	small
wbLGP-1358	wbLGP-558	0.3015406	2352	2352	moderate
wbLGP-1358	wbLGP-658	0.1847120	2352	2352	small
wbLGP-1358	wbLGP-758	0.1378691	2352	2352	small
wbLGP-1358	wbLGP-858	0.2044047	2352	2352	small
wbLGP-1358	wbLGP-958	0.0714880	2352	2352	small
wbLGP-458	wbLGP-558	0.0115092	2352	2352	small
wbLGP-458	wbLGP-658	0.2420159	2352	2352	small
wbLGP-458	wbLGP-758	0.2603953	2352	2352	small
wbLGP-458	wbLGP-858	0.1864424	2352	2352	small
wbLGP-458	wbLGP-958	0.2951134	2352	2352	small
wbLGP-558	wbLGP-658	0.3159288	2352	2352	moderate
wbLGP-558	wbLGP-758	0.3114833	2352	2352	moderate
wbLGP-558	wbLGP-858	0.2210771	2352	2352	small
wbLGP-558	wbLGP-958	0.3280744	2352	2352	moderate
wbLGP-658	wbLGP-758	0.1151963	2352	2352	small
wbLGP-658	wbLGP-858	0.0129152	2352	2352	small
wbLGP-658	wbLGP-958	0.1975321	2352	2352	small
wbLGP-758	wbLGP-858	0.1643324	2352	2352	small
wbLGP-758	wbLGP-958	0.0960455	2352	2352	small
wbLGP-858	wbLGP-958	0.1928274	2352	2352	small

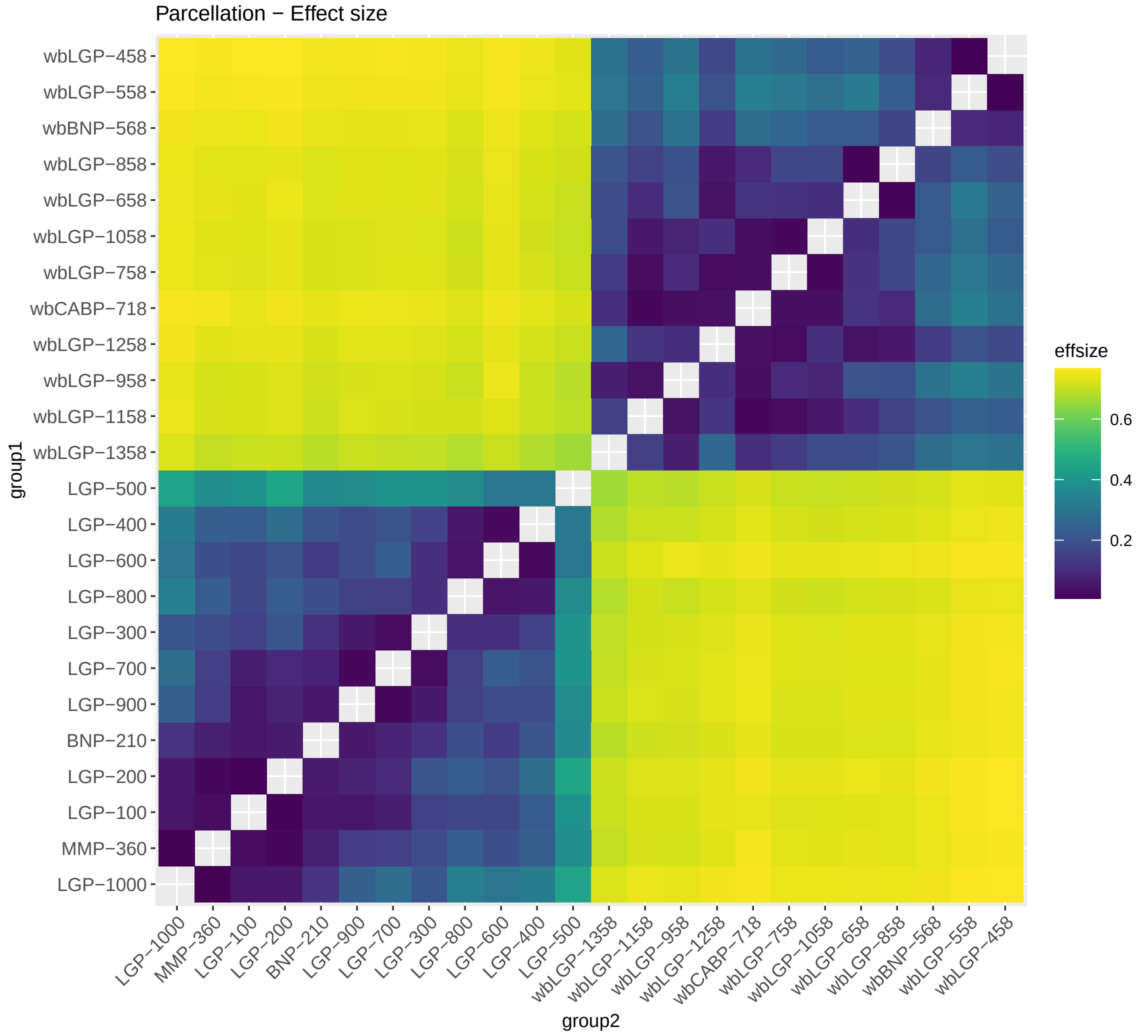

Fig. S6. Parcellation - Effect size

1.12 Variability Changes

Whole Brain vs. Cortex

- Quadrant 1: $V_w \uparrow$,$V_b \uparrow$: 0.0093684
- Quadrant 2: $V_w \downarrow$,$V_b \uparrow$: 0.5999534
- Quadrant 3: $V_w \downarrow$,$V_b \downarrow$: 0.3906782
- Quadrant 4: $V_w \uparrow$,$V_b \downarrow$: 0

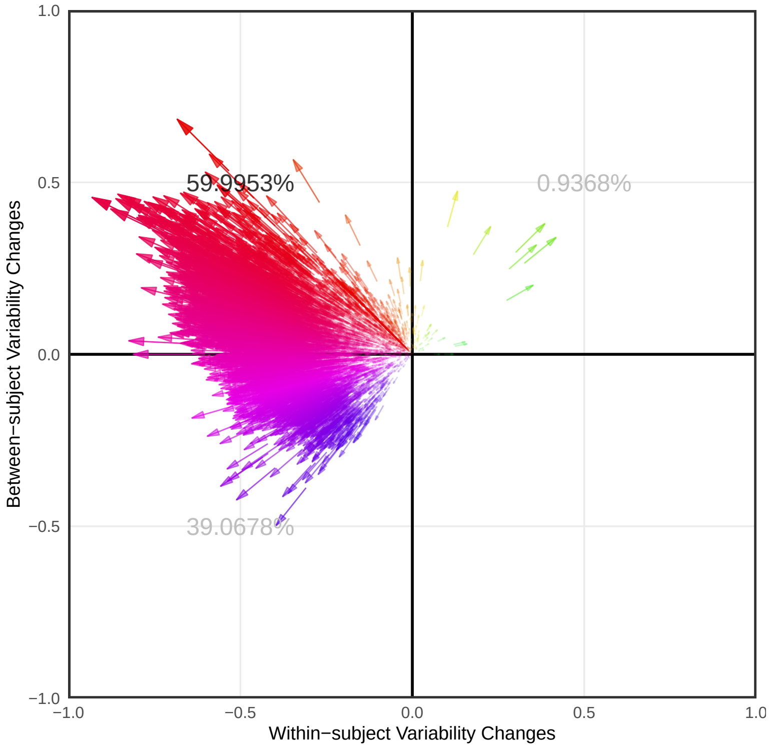

Fig. S7. Parcellation - Variability Changes

2 Frequency Bands - Edge Construction

2.1 ICC Density distribution

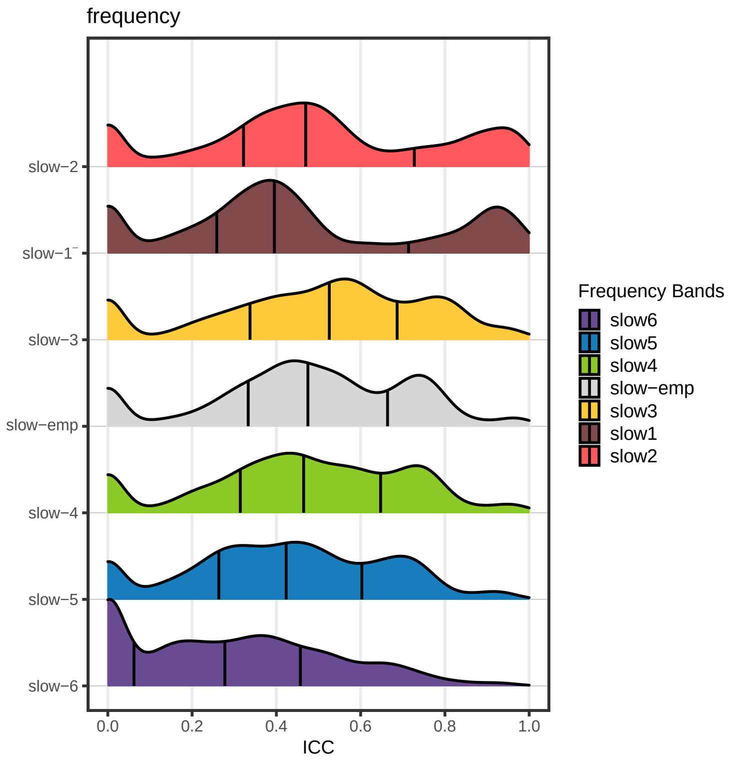

Fig. S8. Frequency - ICC Density distribution

2.2 Almost Perfect (ICCs > 0.8)

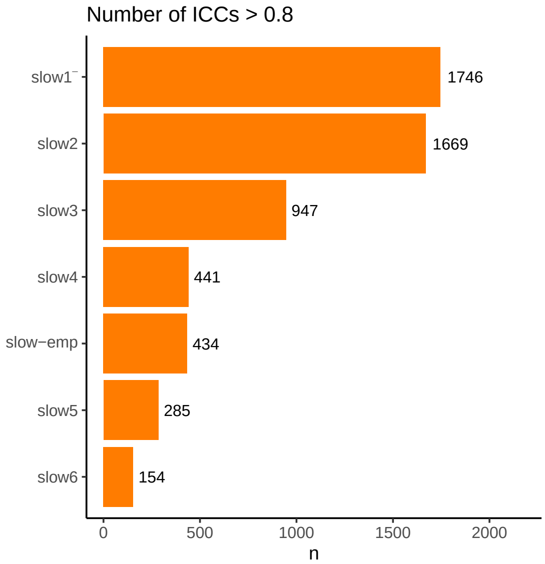

Fig. S9. Frequency - Number of ICC >0.8

2.3 Substantial or Above (ICCs > 0.6)

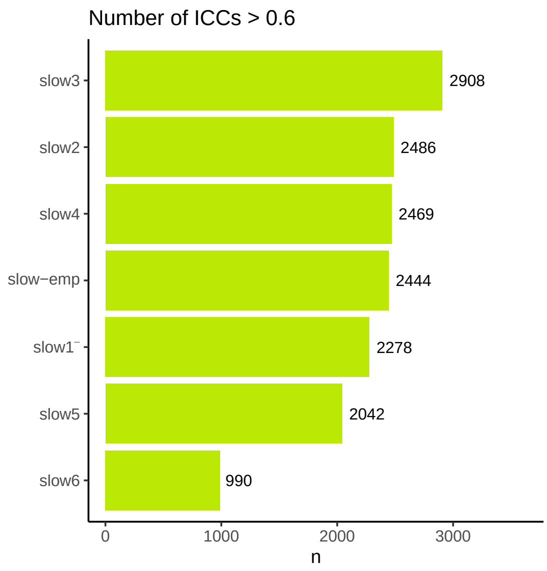

Fig. S10. Frequency - Number of ICC >0.6

2.4 Variability Changes

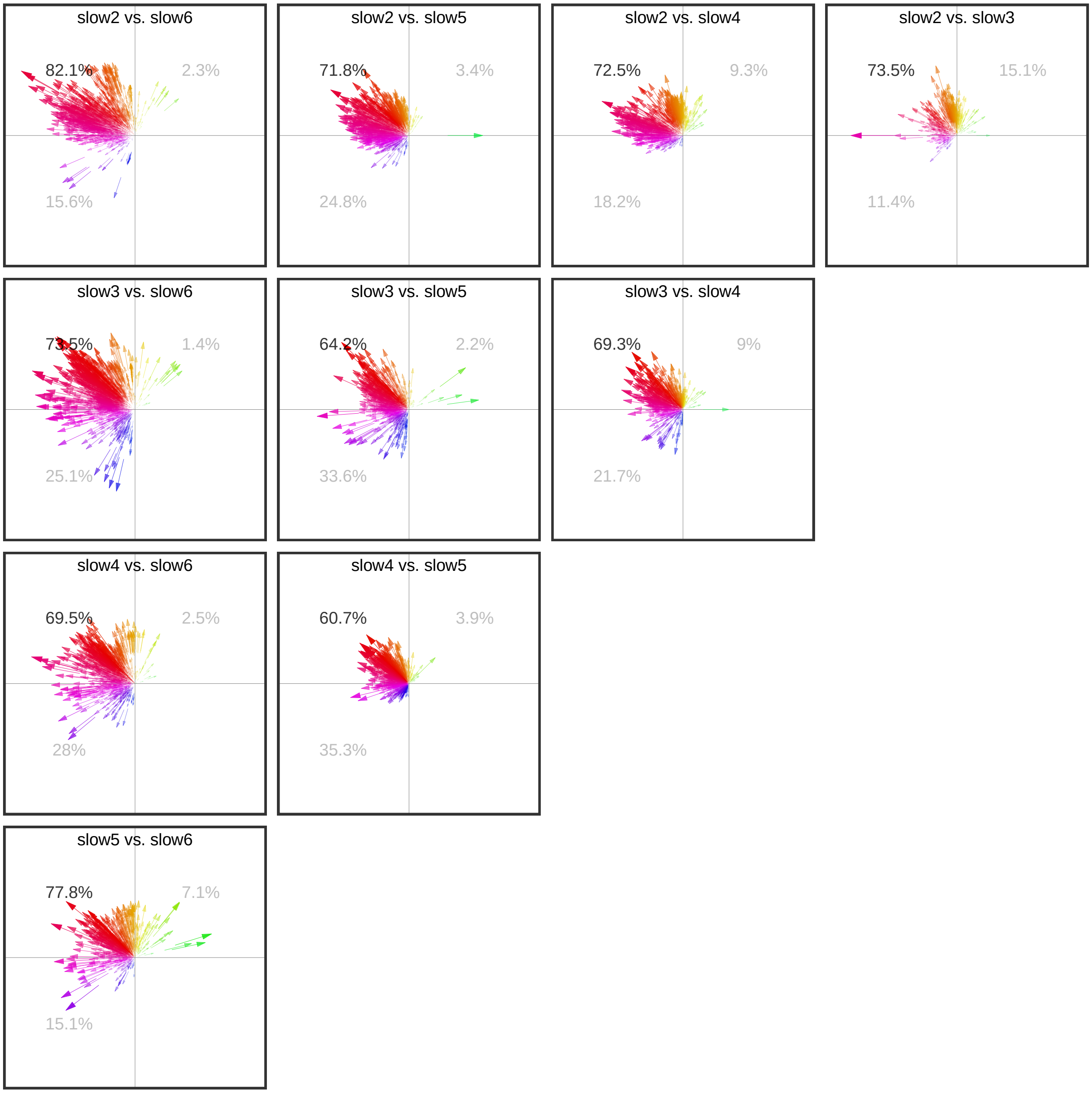

Fig. S11. Frequency - Variability Changes

2.5 Descriptive statistics Mean

Table S9. Frequency - ICC Mean

freq	variable	n	mean	mean_z2r
slow2	ICC.z	8064	0.689	0.5973392
slow1	ICC.z	8064	0.638	0.5635362
slow3	ICC.z	8064	0.621	0.5518239
slow-emp	ICC.z	8064	0.575	0.5190218
slow4	ICC.z	8064	0.560	0.5079774
slow5	ICC.z	8064	0.494	0.4573854
slow6	ICC.z	8064	0.331	0.3194190

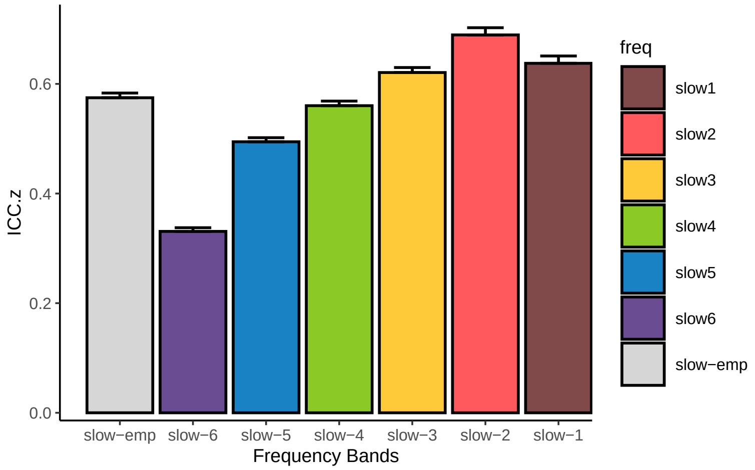

Fig. S12. Frequency - ICC Mean Bar plot

2.6 Descriptive statistics Median

Table S10. Frequency - ICC Median

freq	variable	n	median
slow3	ICC	8064	0.526
slow-emp	ICC	8064	0.475
slow2	ICC	8064	0.470
slow4	ICC	8064	0.465
slow5	ICC	8064	0.423
slow1	ICC	8064	0.395
slow6	ICC	8064	0.278

2.7 Friedman Test

Table S11. Frequency - Friedman Test

.y.	n	statistic	df	p	method
ICC.z	8064	9283.536	6	0	Friedman test

2.8 Friedman Test Effect size

Table S12. Frequency - Friedman Test Effect size

.y.	n	effsize	method	magnitude
ICC.z	8064	0.191872	Kendall W	small

2.9 Paired Wilcoxon signed rank test

Table S13. Frequency - ICC group1 vs. group2

group1	group2	n1	estimate	statistic	alternative	p	p.adj	p.adj.signif
slow-emp	slow1	8064	-0.0162227	14054394	two.sided	9.68e-04	0.0203280	*
slow-emp	slow2	8064	-0.0864131	10505882	two.sided	0.00e+00	0.0000000	****
slow-emp	slow3	8064	-0.0539695	10625450	two.sided	0.00e+00	0.0000000	****
slow-emp	slow4	8064	0.0150546	17102898	two.sided	0.00e+00	0.0000000	****
slow-emp	slow5	8064	0.0841908	23531772	two.sided	0.00e+00	0.0000000	****
slow-emp	slow6	8064	0.2231625	26254891	two.sided	0.00e+00	0.0000000	****
slow1	slow2	8064	-0.0624239	9900068	two.sided	0.00e+00	0.0000000	****
slow1	slow3	8064	-0.0200081	13838087	two.sided	8.77e-05	0.0018417	**
slow1	slow4	8064	0.0325830	15946425	two.sided	0.00e+00	0.0000000	****
slow1	slow5	8064	0.1039074	18945816	two.sided	0.00e+00	0.0000000	****
slow1	slow6	8064	0.2451552	23735075	two.sided	0.00e+00	0.0000000	****
slow2	slow3	8064	0.0367993	16494499	two.sided	0.00e+00	0.0000000	****
slow2	slow4	8064	0.1038401	19454026	two.sided	0.00e+00	0.0000000	****
slow2	slow5	8064	0.1643515	22530367	two.sided	0.00e+00	0.0000000	****
slow2	slow6	8064	0.2906672	25875758	two.sided	0.00e+00	0.0000000	****
slow3	slow4	8064	0.0703217	20070190	two.sided	0.00e+00	0.0000000	****
slow3	slow5	8064	0.1355815	23423580	two.sided	0.00e+00	0.0000000	****
slow3	slow6	8064	0.2698008	26652991	two.sided	0.00e+00	0.0000000	****
slow4	slow5	8064	0.0707338	21276606	two.sided	0.00e+00	0.0000000	****
slow4	slow6	8064	0.2083264	25815007	two.sided	0.00e+00	0.0000000	****
slow5	slow6	8064	0.1410985	24873960	two.sided	0.00e+00	0.0000000	****

2.10 Kullback-leibler divergence Map

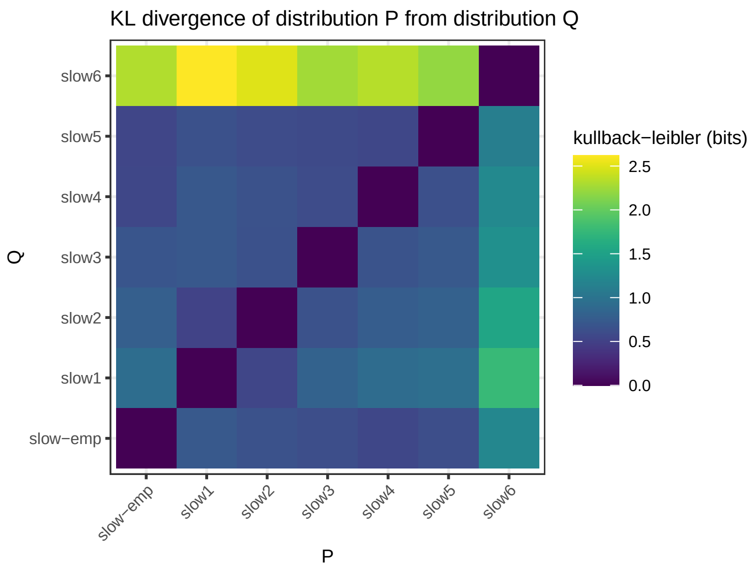

Fig. S13. Frequency - KL divergence Map

2.11 Significance Map

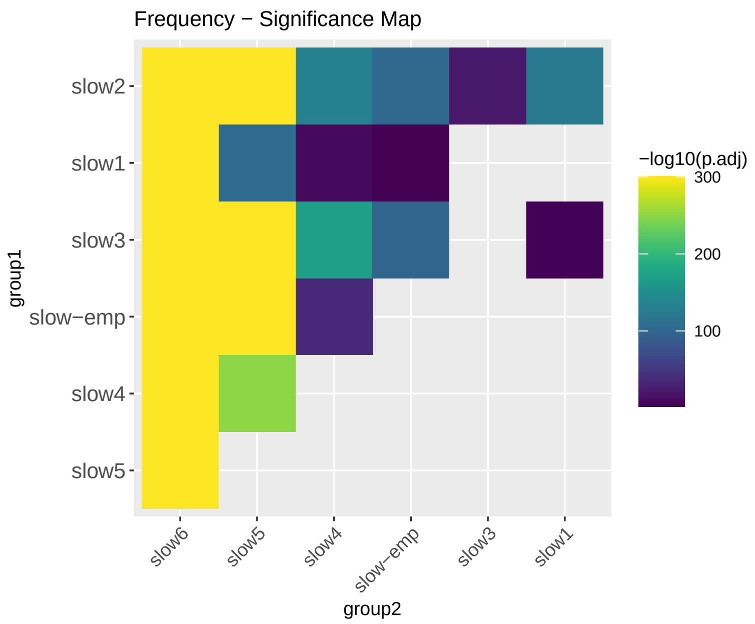

Fig. S14. Frequency - Significance Map

2.12 Effect size

Table S14. Frequency - Effect size

group1	group2	effsize	n1	n2	magnitude
slow-emp	slow1	0.0296722	8064	8064	small
slow-emp	slow2	0.2374189	8064	8064	small
slow-emp	slow3	0.2357191	8064	8064	small
slow-emp	slow4	0.1417849	8064	8064	small
slow-emp	slow5	0.5094890	8064	8064	large
slow-emp	slow6	0.6519342	8064	8064	large
slow1	slow2	0.2699083	8064	8064	small
slow1	slow3	0.0520287	8064	8064	small
slow1	slow4	0.0646878	8064	8064	small
slow1	slow5	0.2408327	8064	8064	small
slow1	slow6	0.5023808	8064	8064	large
slow2	slow3	0.1000050	8064	8064	small
slow2	slow4	0.2699834	8064	8064	small
slow2	slow5	0.4490800	8064	8064	moderate
slow2	slow6	0.6292308	8064	8064	large
slow3	slow4	0.3096116	8064	8064	moderate
slow3	slow5	0.5028784	8064	8064	large
slow3	slow6	0.6802808	8064	8064	large
slow4	slow5	0.3770518	8064	8064	moderate
slow4	slow6	0.6310338	8064	8064	large
slow5	slow6	0.5700624	8064	8064	large

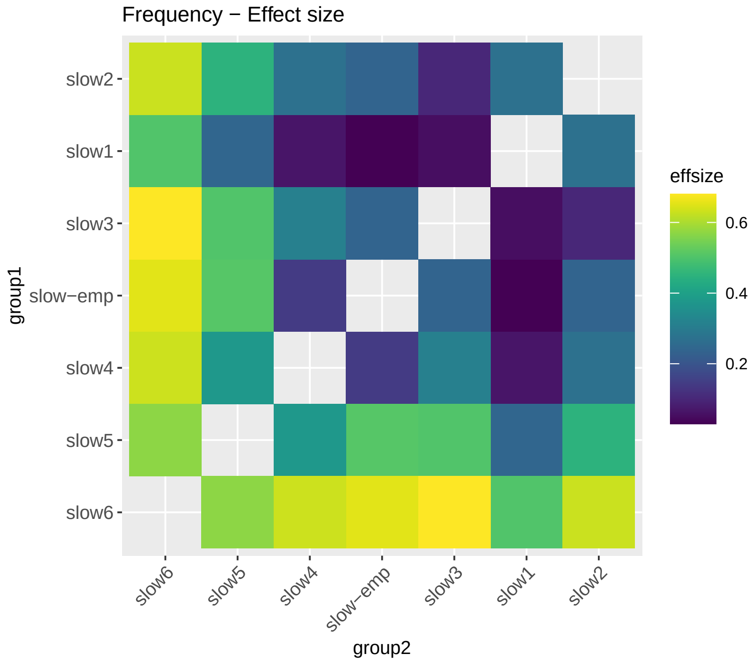

Fig. S15. Frequency - Effect size

3 R Tranforms - Edge Construction

Many network metrics are not well defined for negatively weighted connections. In order to ensure that the connection weights are positive only, we applied four types of transformations to the symmetric correlation matrix: the **positive** (Eq.pos), **absolute** (Eq.abs), **exponential** (Eq.exp) and **distance-inverse** (Eq.div) functions, respectively. This avoids the negative values in the inter-node connectivity matrix $W = (w_{ij})$ where $z_{ij} = \tanh^{-1}(r_{ij})$ is Fisher's z—transformation.

$$w_{ij} = \frac{z_{ij} + |z_{ij}|}{2} \in [0, \infty) \tag{pos}$$

$$w_{ij} = |z_{ij}| \in [0, \infty) \tag{abs}$$

$$w_{ij} = \mathbf{e}^{z_{ij}} \in [0, \infty) \tag{exp}$$

$$w_{ij} = \frac{2}{\sqrt{2 \times (1 - r_{ij})}} \in (0, \infty) \tag{div}$$

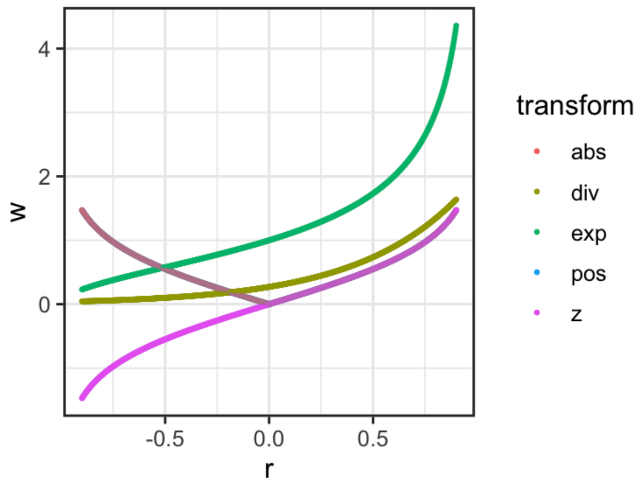

Fig. S16. Transform r to weight

3.1 ICC Density distribution

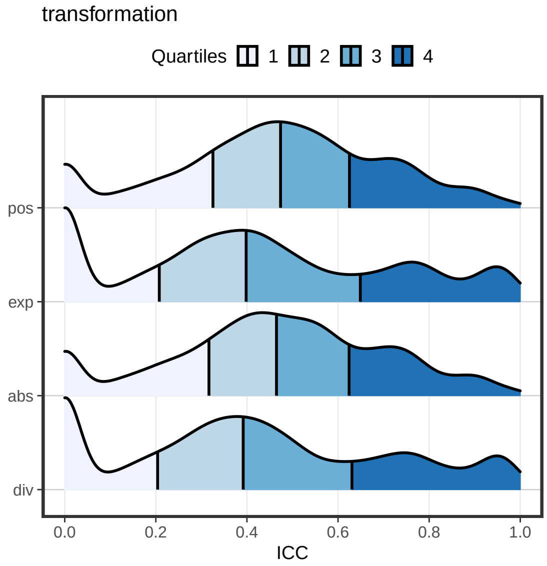

Fig. S17. Transforms - ICC Density distribution

3.2 Almost Perfect (ICCs > 0.8)

Table S15. Transforms - Number of ICCs > 0.8

transform	n
exp	1855
div	1740
abs	1050
pos	1031

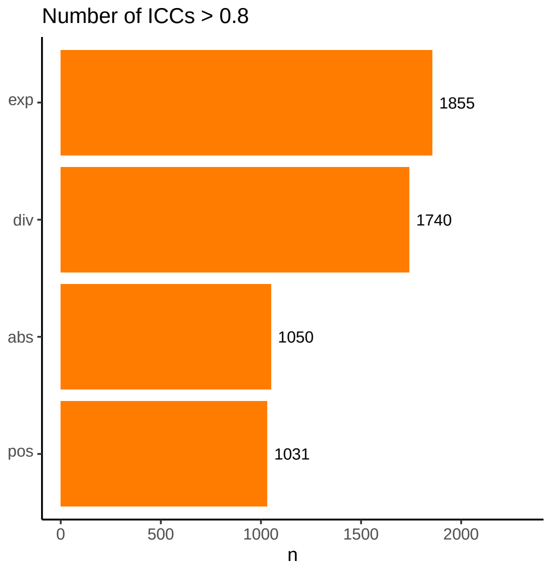

Fig. S18. Transforms - Number of ICC > 0.8

3.3 Substantial or Above (ICCs > 0.6)

Table S16. Transforms - Number of ICCs > 0.6

transform	n
pos	3977
exp	3930
abs	3912
div	3798

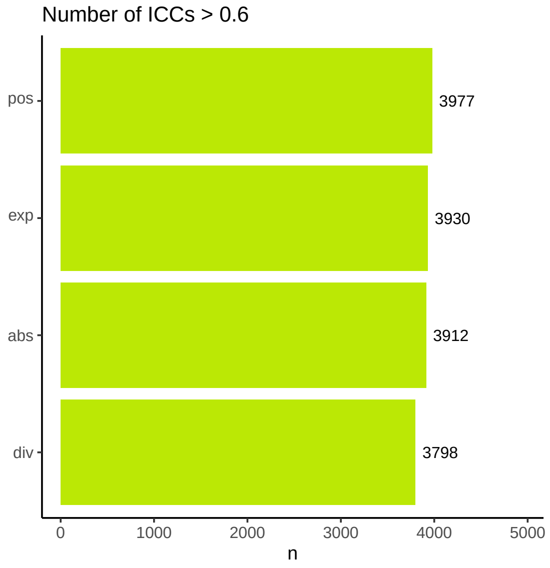

Fig. S19. Transforms - Number of ICC > 0.8

3.4 Descriptive statistics Mean

Table S17. Transforms - ICC Mean

transform	variable	n	mean	mean_z2r
pos	ICC.z	14112	0.564	0.5109392
exp	ICC.z	14112	0.561	0.5087190
abs	ICC.z	14112	0.560	0.5079774
div	ICC.z	14112	0.548	0.4990198

3.5 Descriptive statistics Median

Table S18. Transforms - ICC Median

transform	variable	n	median
pos	ICC	14112	0.474
abs	ICC	14112	0.465
exp	ICC	14112	0.398
div	ICC	14112	0.392

3.6 Friedman Test

Table S19. Transforms - Friedman Test

.y.	n	statistic	df	p	method
ICC.z	14112	480.0639	3	0	Friedman test

3.7 Friedman Test Effect size

Table S20. Transforms - Friedman Test Effect size

.y.	n	effsize	method	magnitude
ICC.z	14112	0.0113394	Kendall W	small

3.8 Paired Wilcoxon signed rank test

Table S21. Transforms - ICC group1 vs. group2

group1	group2	n1	statistic	alternative	p	p.adj	p.adj.signif
abs	div	14112	50949564	two.sided	0e+00	0.0e+00	****
abs	exp	14112	47946915	two.sided	8e-07	4.8e-06	****
abs	pos	14112	44430310	two.sided	3e-03	1.8e-02	*
div	exp	14112	35746898	two.sided	0e+00	0.0e+00	****
div	pos	14112	39669204	two.sided	0e+00	0.0e+00	****
exp	pos	14112	42638630	two.sided	0e+00	0.0e+00	****

3.9 Kullback-leibler divergence Map

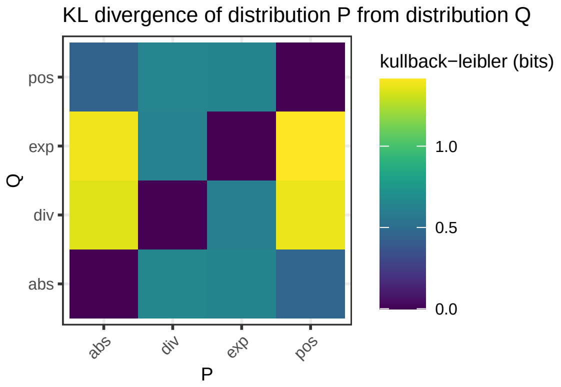

Fig. S20. Transforms - KL divergence Map

3.10 Significance map

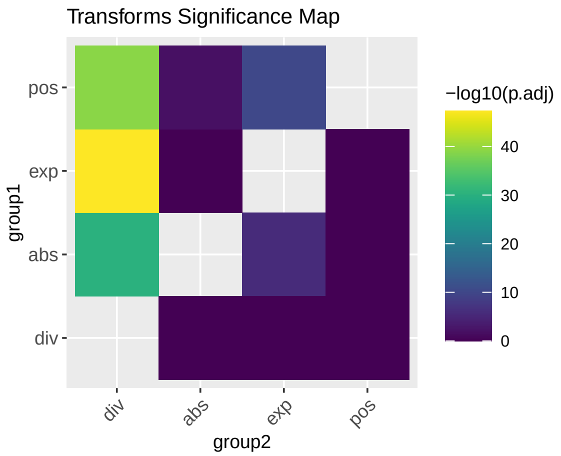

Fig. S21. Transforms - Significance Map

3.11 Effect size

Table S22. Transforms - Effect size

group1	group2	effsize	n1	n2	magnitude
abs	div	0.1009229	14112	14112	small
abs	exp	0.0439603	14112	14112	small
abs	pos	0.0232195	14112	14112	small
div	exp	0.1257376	14112	14112	small
div	pos	0.1139965	14112	14112	small
exp	pos	0.0582916	14112	14112	small

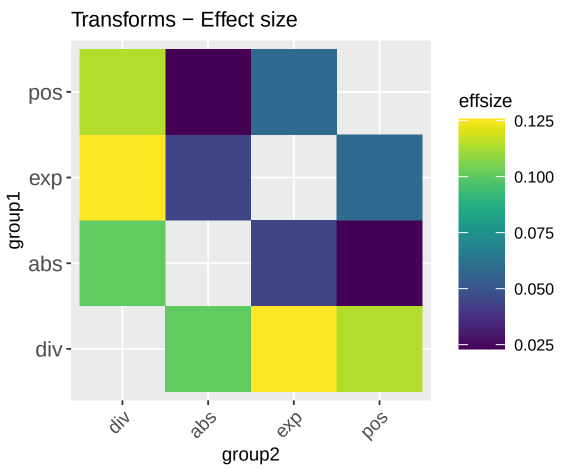

Fig. S22. Transforms - Effect size

4 Schemes - Edge Construction

4.1 ICC Density distribution

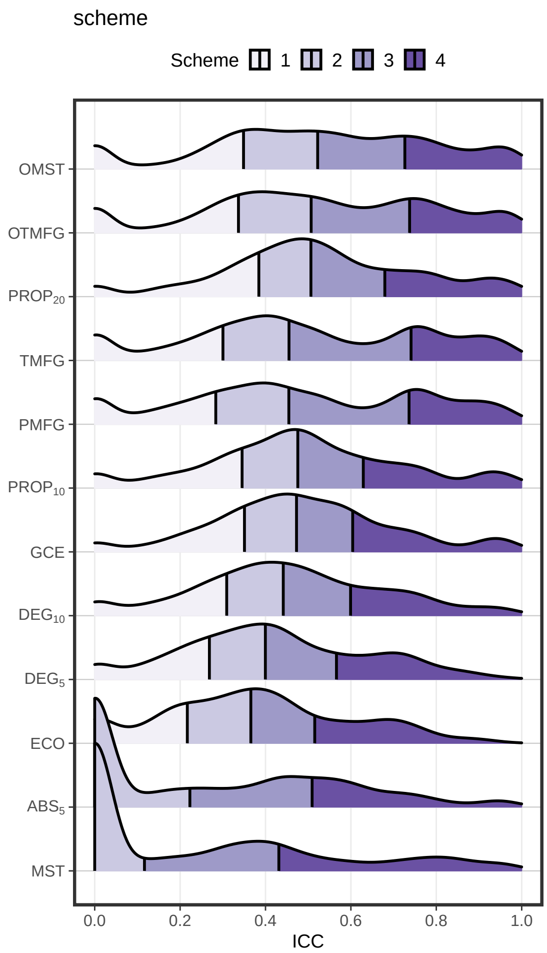

Fig. S23. Schemes - ICC Density distribution

4.2 Almost Perfect (ICCs > 0.8)

Table S23. Schemes - Number of ICCs > 0.8

scheme	n
OTMFG	781
TMFG	767
OMST	765
PMFG	737
$PROP_{20}$	632
$PROP_{10}$	445
MST	362
GCE	352
DEG_{15}	306
DEG_{5}	213
ABS_{05}	189
ECO	127

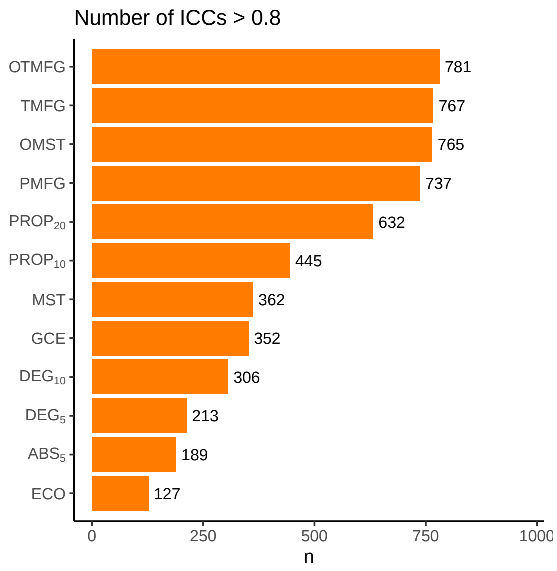

Fig. S24. Schemes - Number of ICC > 0.8

4.3 Substantial or Above (ICCs > 0.6)

Table S24. Schemes - Number of ICCs > 0.8

scheme	n
OMST	1860
OTMFG	1832
TMFG	1670
PMFG	1659
PROP_{20}	1564
PROP_{10}	1345
GCE	1210
DEG_{15}	1175
DEG_{5}	1035
ECO	837
MST	719
ABS_{05}	711

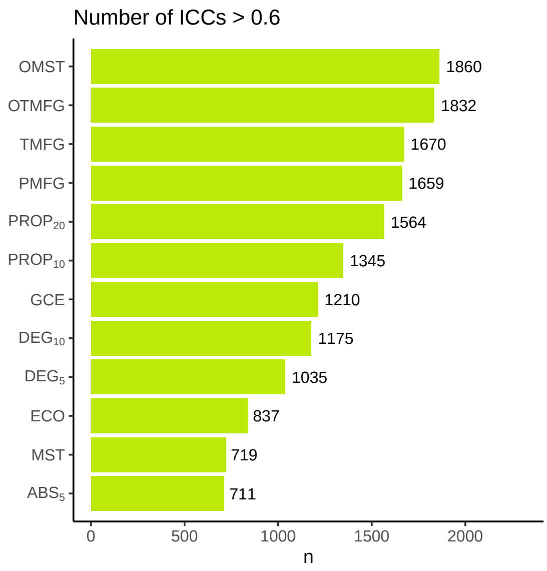

Fig. S25. Schemes - Number of ICC > 0.6

4.4 Varibility Changes

OMST, OTMFG, PROP_{20}, TMFG, PMFG, PROP_{10}

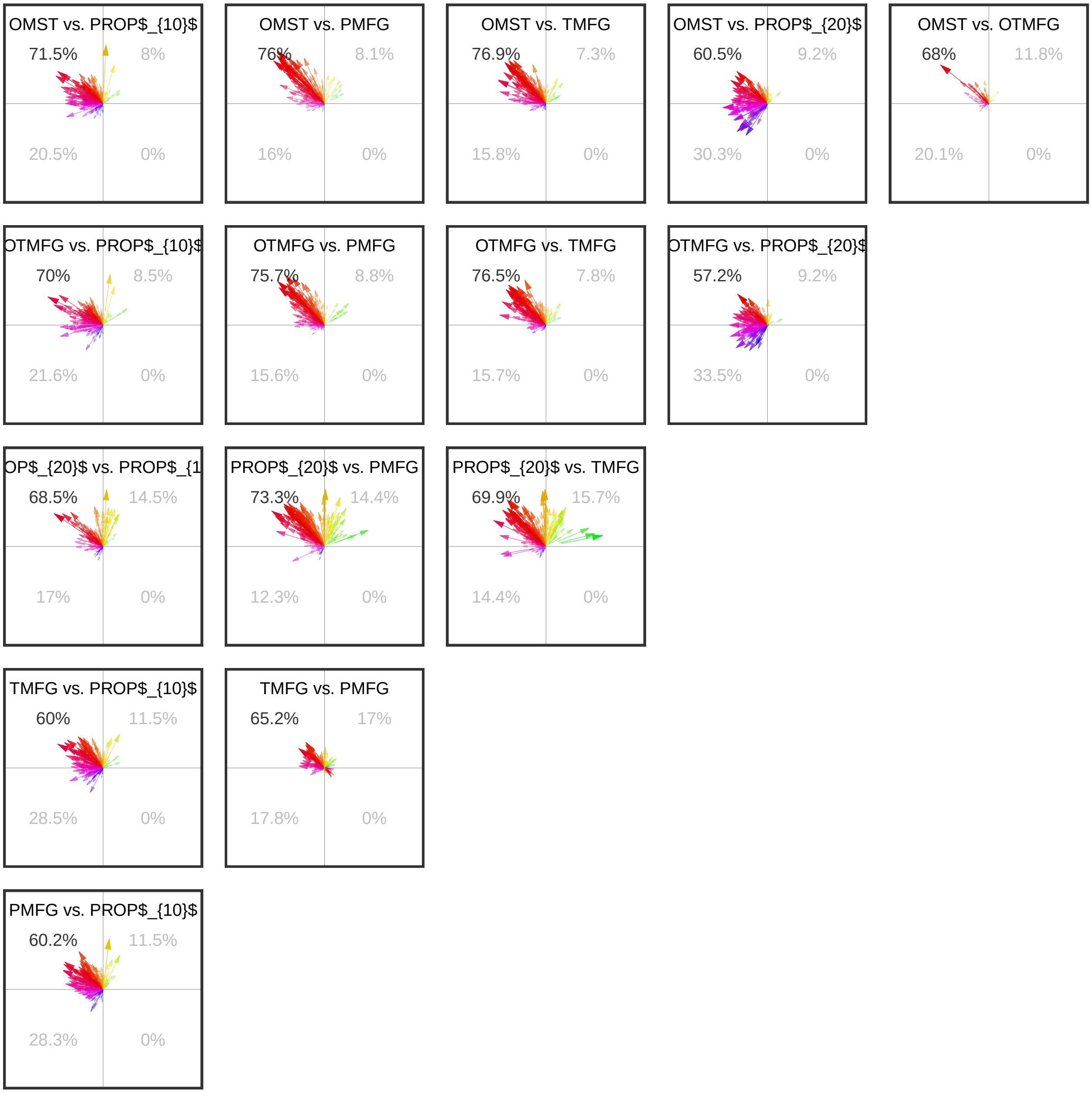

Fig. S26. Schemes - Variability Changes

4.5 Descriptive statistics Mean

Table S25. Schemes - ICC Mean

scheme	variable	n	mean	meanz2r
OMST	ICC.z	4704	0.706	0.6081624
OTMFG	ICC.z	4704	0.697	0.6024601
PROP_{20}	ICC.z	4704	0.683	0.5934662
TMFG	ICC.z	4704	0.647	0.5696469
PMFG	ICC.z	4704	0.635	0.5614855
PROP_{10}	ICC.z	4704	0.617	0.5490358
GCE	ICC.z	4704	0.595	0.5334821
DEG_{15}	ICC.z	4704	0.547	0.4982684
DEG_{5}	ICC.z	4704	0.486	0.4510359
ECO	ICC.z	4704	0.431	0.4061567
ABS_{05}	ICC.z	4704	0.337	0.3247965
MST	ICC.z	4704	0.319	0.3086024

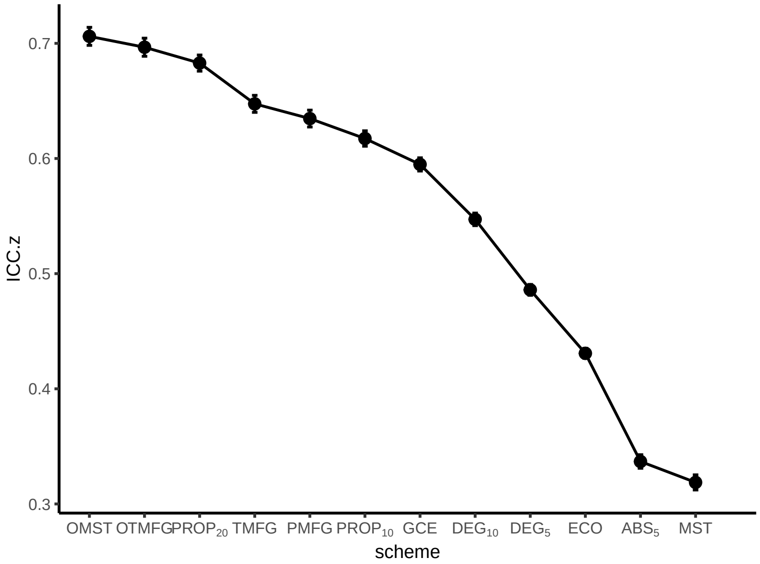

Fig. S27. Schemes - ICC mean and se

4.6 Descriptive statistics Median

Table S26. Schemes - ICC Median

scheme	variable	n	median
OMST	ICC	4704	0.522
OTMFG	ICC	4704	0.507
$PROP_{20}$	ICC	4704	0.506
$PROP_{10}$	ICC	4704	0.476
GCE	ICC	4704	0.473
PMFG	ICC	4704	0.455
TMFG	ICC	4704	0.455
DEG_{15}	ICC	4704	0.442
DEG_{5}	ICC	4704	0.400
ECO	ICC	4704	0.366
ABS_{05}	ICC	4704	0.223
MST	ICC	4704	0.117

4.7 Friedman Test

Table S27. Schemes - Friedman Test

.y.	n	statistic	df	p	method
ICC.z	4704	9784.317	11	0	Friedman test

4.8 Friedman Test Effect size

Table S28. Schemes - Friedman Test Effect size

.y.	n	effsize	method	magnitude
ICC.z	4704	0.1890909	Kendall W	small

4.9 Paired Wilcoxon signed rank test

Table S29. Schemes - ICC group1 vs. group2

group1	group2	n1	statistic	alternative	p	p.adj	p.adj.signif
ABS_{05}	DEG_{15}	4704	2694254	two.sided	0.00e+00	0.0000000	****
ABS_{05}	DEG_{5}	4704	3270500	two.sided	0.00e+00	0.0000000	****
ABS_{05}	ECO	4704	3943115	two.sided	0.00e+00	0.0000000	****
ABS_{05}	GCE	4704	2271646	two.sided	0.00e+00	0.0000000	****
ABS_{05}	MST	4704	4965999	two.sided	0.00e+00	0.0000003	****
ABS_{05}	OMST	4704	1467195	two.sided	0.00e+00	0.0000000	****
ABS_{05}	OTMFG	4704	1591487	two.sided	0.00e+00	0.0000000	****
ABS_{05}	PMFG	4704	2360874	two.sided	0.00e+00	0.0000000	****

Table S29. Schemes - ICC group1 vs. group2 *(continued)*

group1	group2	n1	statistic	alternative	p	p.adj	p.adj.signif
ABS$_{05}$	PROP$_{10}$	4704	2285372	two.sided	0.00e+00	0.0000000	****
ABS$_{05}$	PROP$_{20}$	4704	1672112	two.sided	0.00e+00	0.0000000	****
ABS$_{05}$	TMFG	4704	2232779	two.sided	0.00e+00	0.0000000	****
DEG$_{15}$	DEG$_{5}$	4704	7803402	two.sided	0.00e+00	0.0000000	****
DEG$_{15}$	ECO	4704	8444214	two.sided	0.00e+00	0.0000000	****
DEG$_{15}$	GCE	4704	4108796	two.sided	0.00e+00	0.0000000	****
DEG$_{15}$	MST	4704	8200729	two.sided	0.00e+00	0.0000000	****
DEG$_{15}$	OMST	4704	2665961	two.sided	0.00e+00	0.0000000	****
DEG$_{15}$	OTMFG	4704	2810801	two.sided	0.00e+00	0.0000000	****
DEG$_{15}$	PMFG	4704	4054470	two.sided	0.00e+00	0.0000000	****
DEG$_{15}$	PROP$_{10}$	4704	3445204	two.sided	0.00e+00	0.0000000	****
DEG$_{15}$	PROP$_{20}$	4704	2504887	two.sided	0.00e+00	0.0000000	****
DEG$_{15}$	TMFG	4704	3834624	two.sided	0.00e+00	0.0000000	****
DEG$_{5}$	ECO	4704	8040590	two.sided	0.00e+00	0.0000000	****
DEG$_{5}$	GCE	4704	3328222	two.sided	0.00e+00	0.0000000	****
DEG$_{5}$	MST	4704	7483490	two.sided	0.00e+00	0.0000000	****
DEG$_{5}$	OMST	4704	2135420	two.sided	0.00e+00	0.0000000	****
DEG$_{5}$	OTMFG	4704	2269997	two.sided	0.00e+00	0.0000000	****
DEG$_{5}$	PMFG	4704	2996624	two.sided	0.00e+00	0.0000000	****
DEG$_{5}$	PROP$_{10}$	4704	2832475	two.sided	0.00e+00	0.0000000	****
DEG$_{5}$	PROP$_{20}$	4704	1965712	two.sided	0.00e+00	0.0000000	****
DEG$_{5}$	TMFG	4704	2747205	two.sided	0.00e+00	0.0000000	****
ECO	GCE	4704	2438845	two.sided	0.00e+00	0.0000000	****
ECO	MST	4704	6602202	two.sided	0.00e+00	0.0000000	****
ECO	OMST	4704	1445475	two.sided	0.00e+00	0.0000000	****
ECO	OTMFG	4704	1561756	two.sided	0.00e+00	0.0000000	****
ECO	PMFG	4704	2072641	two.sided	0.00e+00	0.0000000	****
ECO	PROP$_{10}$	4704	2273516	two.sided	0.00e+00	0.0000000	****
ECO	PROP$_{20}$	4704	1494010	two.sided	0.00e+00	0.0000000	****
ECO	TMFG	4704	1864579	two.sided	0.00e+00	0.0000000	****
GCE	MST	4704	8955851	two.sided	0.00e+00	0.0000000	****
GCE	OMST	4704	3276387	two.sided	0.00e+00	0.0000000	****
GCE	OTMFG	4704	3459105	two.sided	0.00e+00	0.0000000	****
GCE	PMFG	4704	5127650	two.sided	2.85e-05	0.0018810	**
GCE	PROP$_{10}$	4704	5081947	two.sided	3.30e-06	0.0002158	***
GCE	PROP$_{20}$	4704	3034877	two.sided	0.00e+00	0.0000000	****
GCE	TMFG	4704	4875617	two.sided	0.00e+00	0.0000000	****
MST	OMST	4704	712172	two.sided	0.00e+00	0.0000000	****
MST	OTMFG	4704	795758	two.sided	0.00e+00	0.0000000	****
MST	PMFG	4704	1340430	two.sided	0.00e+00	0.0000000	****
MST	PROP$_{10}$	4704	1771350	two.sided	0.00e+00	0.0000000	****
MST	PROP$_{20}$	4704	1149079	two.sided	0.00e+00	0.0000000	****
MST	TMFG	4704	1164787	two.sided	0.00e+00	0.0000000	****
OMST	OTMFG	4704	5863222	two.sided	0.00e+00	0.0000000	****
OMST	PMFG	4704	7183474	two.sided	0.00e+00	0.0000000	****
OMST	PROP$_{10}$	4704	7386941	two.sided	0.00e+00	0.0000000	****
OMST	PROP$_{20}$	4704	6196410	two.sided	0.00e+00	0.0000000	****

Table S29. Schemes - ICC group1 vs. group2 *(continued)*

group1	group2	n1	statistic	alternative	p	p.adj	p.adj.signif
OMST	TMFG	4704	6887505	two.sided	0.00e+00	0.0000000	****
OTMFG	PMFG	4704	7021466	two.sided	0.00e+00	0.0000000	****
OTMFG	$PROP_{10}$	4704	7154543	two.sided	0.00e+00	0.0000000	****
OTMFG	$PROP_{20}$	4704	5995825	two.sided	0.00e+00	0.0000028	****
OTMFG	TMFG	4704	6682826	two.sided	0.00e+00	0.0000000	****
PMFG	$PROP_{10}$	4704	5618652	two.sided	1.45e-01	1.0000000	ns
PMFG	$PROP_{20}$	4704	4515273	two.sided	0.00e+00	0.0000000	****
PMFG	TMFG	4704	4119850	two.sided	0.00e+00	0.0000000	****
$PROP_{10}$	$PROP_{20}$	4704	2886243	two.sided	0.00e+00	0.0000000	****
$PROP_{10}$	TMFG	4704	5169273	two.sided	4.31e-04	0.0284460	*
$PROP_{20}$	TMFG	4704	6289407	two.sided	0.00e+00	0.0000000	****

4.10 Kullback-leibler divergence Map

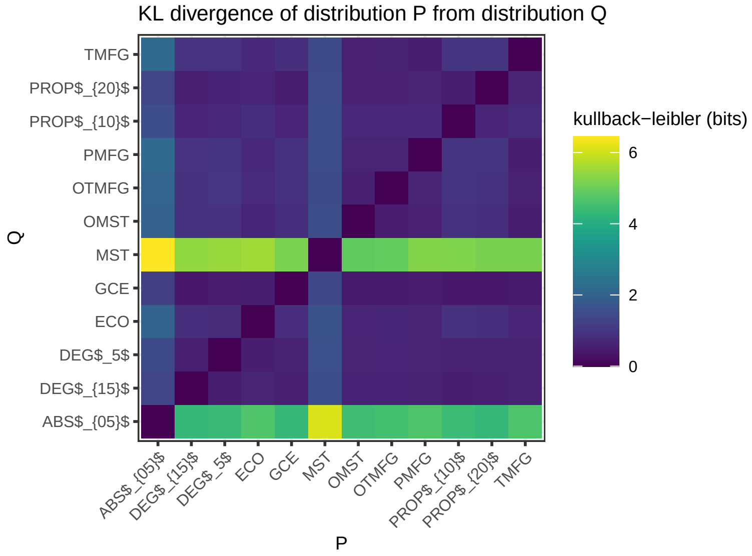

Fig. S28. Schemes - KL divergence Map

4.11 Significance map

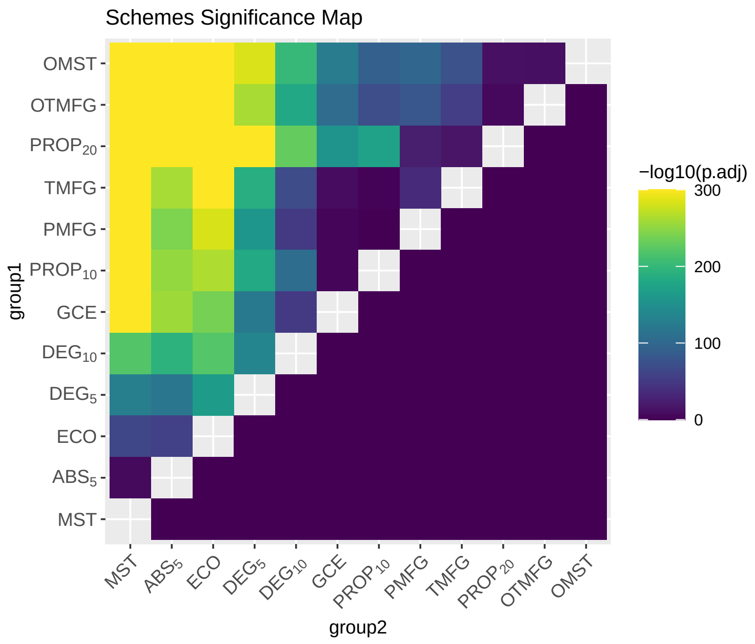

Fig. S29. Schemes - Significance Map

4.12 Effect size

Table S30. Schemes - Effect size

group1	group2	effsize	n1	n2	magnitude
ABS_{05}	DEG_{15}	0.4336085	4704	4704	moderate
ABS_{05}	DEG_{5}	0.3385623	4704	4704	moderate
ABS_{05}	ECO	0.2339104	4704	4704	small
ABS_{05}	GCE	0.5006219	4704	4704	large
ABS_{05}	MST	0.1010940	4704	4704	small
ABS_{05}	OMST	0.6272196	4704	4704	large
ABS_{05}	OTMFG	0.6064118	4704	4704	large
ABS_{05}	PMFG	0.4861088	4704	4704	moderate
ABS_{05}	PROP_{10}	0.4962328	4704	4704	moderate
ABS_{05}	PROP_{20}	0.5953801	4704	4704	large
ABS_{05}	TMFG	0.5043800	4704	4704	large
DEG_{15}	DEG_{5}	0.3656085	4704	4704	moderate
DEG_{15}	ECO	0.4643143	4704	4704	moderate
DEG_{15}	GCE	0.2213037	4704	4704	small
DEG_{15}	MST	0.4629302	4704	4704	moderate
DEG_{15}	OMST	0.4439416	4704	4704	moderate
DEG_{15}	OTMFG	0.4196640	4704	4704	moderate
DEG_{15}	PMFG	0.2243101	4704	4704	small
DEG_{15}	PROP_{10}	0.3230340	4704	4704	moderate

Table S30. Schemes - Effect size *(continued)*

group1	group2	effsize	n1	n2	magnitude
DEG_{15}	$PROP_{20}$	0.4729914	4704	4704	moderate
DEG_{15}	TMFG	0.2592393	4704	4704	small
DEG_5	ECO	0.4011082	4704	4704	moderate
DEG_5	GCE	0.3443975	4704	4704	moderate
DEG_5	MST	0.3530459	4704	4704	moderate
DEG_5	OMST	0.5266713	4704	4704	large
DEG_5	OTMFG	0.5058619	4704	4704	large
DEG_5	PMFG	0.3921309	4704	4704	moderate
DEG_5	$PROP_{10}$	0.4209417	4704	4704	moderate
DEG_5	$PROP_{20}$	0.5574678	4704	4704	large
DEG_5	TMFG	0.4294553	4704	4704	moderate
ECO	GCE	0.4829184	4704	4704	moderate
ECO	MST	0.2472343	4704	4704	small
ECO	OMST	0.6246376	4704	4704	large
ECO	OTMFG	0.6073007	4704	4704	large
ECO	PMFG	0.5251605	4704	4704	large
ECO	$PROP_{10}$	0.5082960	4704	4704	large
ECO	$PROP_{20}$	0.6300488	4704	4704	large
ECO	TMFG	0.5579538	4704	4704	large
GCE	MST	0.5682424	4704	4704	large
GCE	OMST	0.3497301	4704	4704	moderate
GCE	OTMFG	0.3209678	4704	4704	moderate
GCE	PMFG	0.0609041	4704	4704	small
GCE	$PROP_{10}$	0.0677587	4704	4704	small
GCE	$PROP_{20}$	0.3900709	4704	4704	moderate
GCE	TMFG	0.0968897	4704	4704	small
MST	OMST	0.7314210	4704	4704	large
MST	OTMFG	0.7144886	4704	4704	large
MST	PMFG	0.6232274	4704	4704	large
MST	$PROP_{10}$	0.5694680	4704	4704	large
MST	$PROP_{20}$	0.6742707	4704	4704	large
MST	TMFG	0.6506873	4704	4704	large
OMST	OTMFG	0.1072281	4704	4704	small
OMST	PMFG	0.3108939	4704	4704	moderate
OMST	$PROP_{10}$	0.2977166	4704	4704	small
OMST	$PROP_{20}$	0.1111673	4704	4704	small
OMST	TMFG	0.2713060	4704	4704	small
OTMFG	PMFG	0.2809687	4704	4704	small
OTMFG	$PROP_{10}$	0.2638212	4704	4704	small
OTMFG	$PROP_{20}$	0.0798667	4704	4704	small
OTMFG	TMFG	0.2305216	4704	4704	small

Table S30. Schemes - Effect size *(continued)*

group1	group2	effsize	n1	n2	magnitude
PMFG	$PROP_{10}$	0.0210514	4704	4704	small
PMFG	$PROP_{20}$	0.1549509	4704	4704	small
PMFG	TMFG	0.1861855	4704	4704	small
$PROP_{10}$	$PROP_{20}$	0.4119006	4704	4704	moderate
$PROP_{10}$	TMFG	0.0511920	4704	4704	small
$PROP_{20}$	TMFG	0.1263285	4704	4704	small

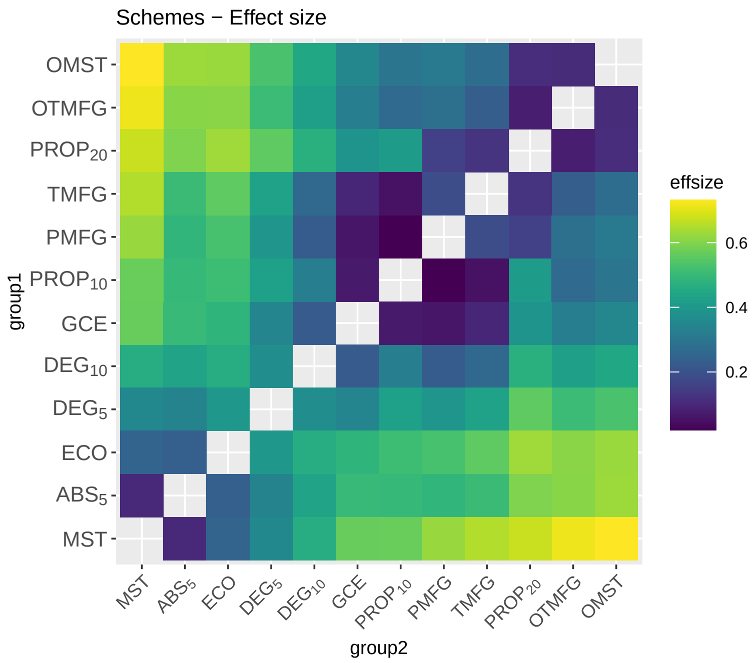

Fig. S30. Schemes - Effect size

5 Metrics - Network Analysis

5.1 Metrics

Superscript b (eg. Eg^b) is used for the binary graphs, superscript w (eg. Eg^w) is used for the weighted graphs, and superscript n (eg. Eg^n) is used for the normalized weighted graphs.

Table S31. Brief descriptions of the network metrics examined

Level	Measure	Attribute	Character
Global	basic	degree	k
		number of edge	n_e
		density	d
	integration	global efficiency	Eg
		average shortest path length	Lp_a
		average nodal path length	Lp_b
		pseudo diameter	D
	segregation	clusteringcoef by BCT toolbox	Cp_a
		clusteringcoef by Graph-tool	Cp_b
		local efficiency algorithm 1 by BCT toolbox	$Eloc_1$
		local efficiency algorithm 2 by BCT toolbox	$Eloc_2$
		modularity	Q
		transitivity by BCT toolbox	Tr_a
		transitivity by Graph-tool	Tr_b
	centrality	average betweenness	Bc
		average eigenvector	Ec
		average pagerank	Pc
		average subgraph	Sc
	resilience	assortativity	r
		scalar assortativity	r_s
		synchronizability	S
		average resolvent	Rv
Nodal	integration	nodal path length	Lp_a
		local path length	Lp_b
	segregation	clustering coefficient by BCT toolbox	Cp_a
		clustering coefficient Graph-tool	Cp_b
		local efficiency 1 by BCT toolbox	$Eloc_1$
		local efficiency 2 by BCT toolbox	$Eloc_2$
		nodal efficiency	E_{nodal}
	centrality	degree centrality	Dc
		betweeness centrality	Bc
		eigenvector centrality	Ec
		pagerank centrality	Pc
		subgraph centrality	Sc
		resolvent centrality	Rc

5.2 ICC Density distribution

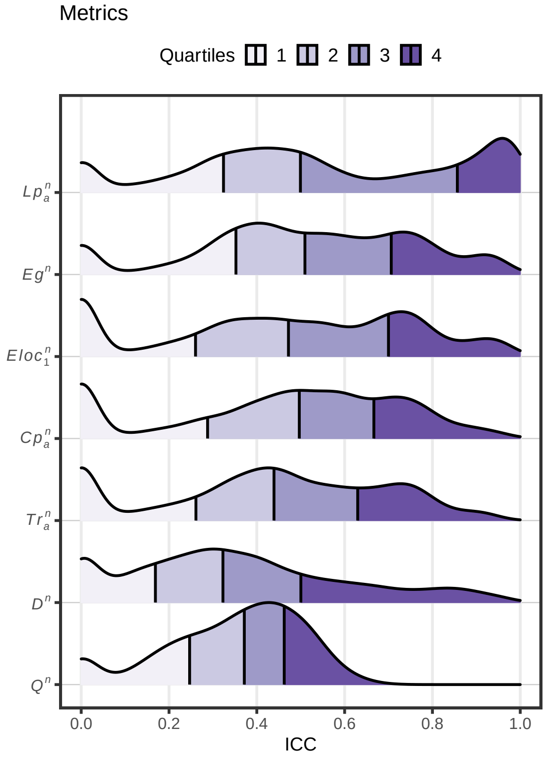

Fig. S31. Metrics - Density distribution

5.3 Almost Perfect (ICCs > 0.8)

Table S32. Metrics - Number of ICCs > 0.8

	character	n
3	Lp_a^n	2350
4	Eg^n	924
2	$Eloc_1^n$	835
1	Cp_a^n	575
5	D^n	559
6	Tr_a^n	433

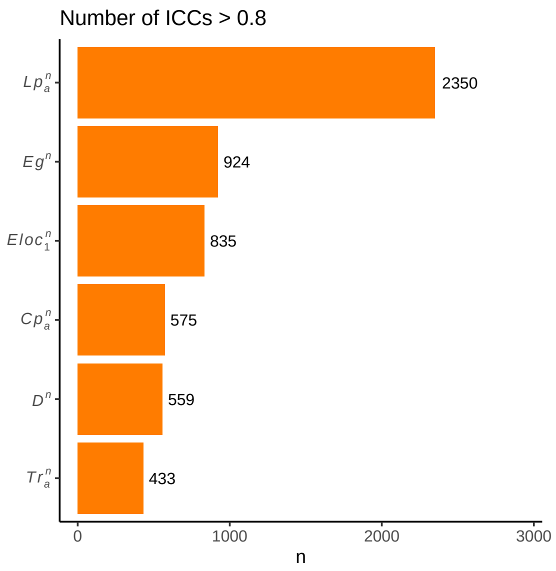

Fig. S32. Metrics - Number of ICC > 0.8

5.4 Substantial or Above (ICCs > 0.6)

Table S33. Metrics - Number of ICCs > 0.6

	character	n
3	Lp_a^n	3194
4	Eg^n	3032
2	$Eloc_1^n$	2886
1	Cp_a^n	2709
7	Tr_a^n	2273
6	D^n	1383
5	Q^n	140

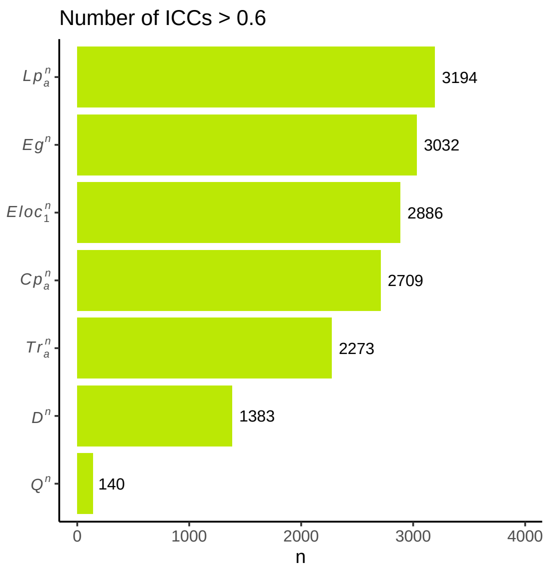

Fig. S33. Metrics - Number of ICC > 0.6

5.5 Descriptive statistics Mean

Table S34. Metrics - ICC Mean

character	n	meanz2r
Lp_a^n	8064	0.6847483
Eg^n	8064	0.5628534
$Eloc_1^n$	8064	0.5241176
Cp_a^n	8064	0.5057482
Tr_a^n	8064	0.4683854
D^n	8064	0.3952442
Q^n	8064	0.3531167

5.6 Descriptive statistics Median

Table S35. Metrics - ICC Median

character	n	median
Eg^n	8064	0.510
Lp_a^n	8064	0.499
Cp_a^n	8064	0.497
$Eloc_1^n$	8064	0.472
Tr_a^n	8064	0.439
Q^n	8064	0.371
D^n	8064	0.323

5.7 Paired Wilcoxon signed rank test

Table S36. Metrics - ICC group1 vs. group2

character1	character2	n1	statistic	alternative	p	p.adj	p.adj.signif
Cp_a^n	$Eloc_1^n$	8064	12227711	two.sided	9.20e-06	0.0001934	***
Cp_a^n	Lp_a^n	8064	8726636	two.sided	0.00e+00	0.0000000	****
Cp_a^n	Eg^n	8064	13406103	two.sided	0.00e+00	0.0000000	****
Cp_a^n	Q^n	8064	23845905	two.sided	0.00e+00	0.0000000	****
Cp_a^n	D^n	8064	23503271	two.sided	0.00e+00	0.0000000	****
Cp_a^n	Tr_a^n	8064	18879595	two.sided	0.00e+00	0.0000000	****
$Eloc_1^n$	Lp_a^n	8064	8214982	two.sided	0.00e+00	0.0000000	****
$Eloc_1^n$	Eg^n	8064	13804539	two.sided	0.00e+00	0.0000000	****
$Eloc_1^n$	Q^n	8064	23296271	two.sided	0.00e+00	0.0000000	****
$Eloc_1^n$	D^n	8064	24208558	two.sided	0.00e+00	0.0000000	****
$Eloc_1^n$	Tr_a^n	8064	17771904	two.sided	0.00e+00	0.0000000	****
Lp_a^n	Eg^n	8064	21277439	two.sided	0.00e+00	0.0000000	****
Lp_a^n	Q^n	8064	26286171	two.sided	0.00e+00	0.0000000	****
Lp_a^n	D^n	8064	28471330	two.sided	0.00e+00	0.0000000	****
Lp_a^n	Tr_a^n	8064	24806988	two.sided	0.00e+00	0.0000000	****
Eg^n	Q^n	8064	26666127	two.sided	0.00e+00	0.0000000	****
Eg^n	D^n	8064	27744888	two.sided	0.00e+00	0.0000000	****
Eg^n	Tr_a^n	8064	21280096	two.sided	0.00e+00	0.0000000	****
Q^n	D^n	8064	15808230	two.sided	3.65e-01	1.0000000	ns
Q^n	Tr_a^n	8064	9856527	two.sided	0.00e+00	0.0000000	****
D^n	Tr_a^n	8064	8856878	two.sided	0.00e+00	0.0000000	****

5.8 Kullback-leibler divergence Map

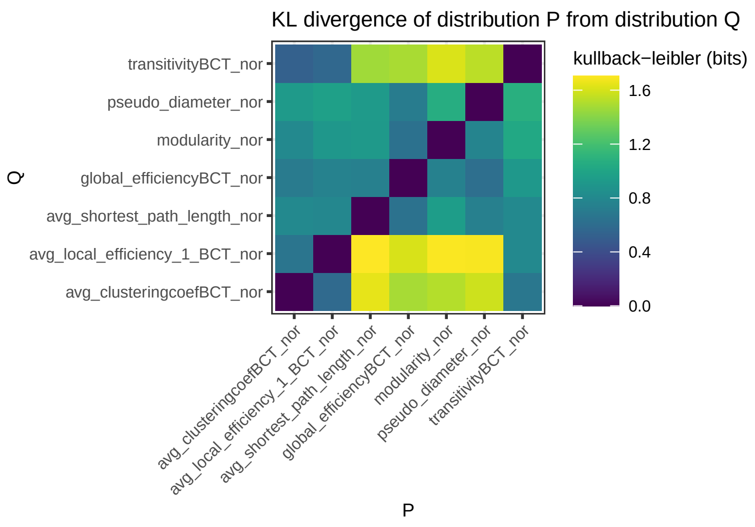

Fig. S34. Metrics - KL divergence Map

5.9 Significance map

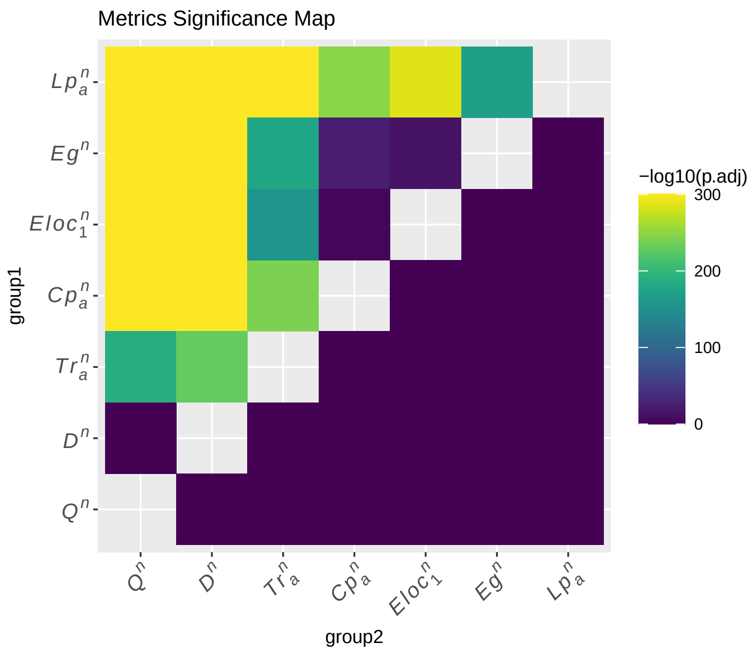

Fig. S35. Metrics - Significance Map

5.10 Effect size

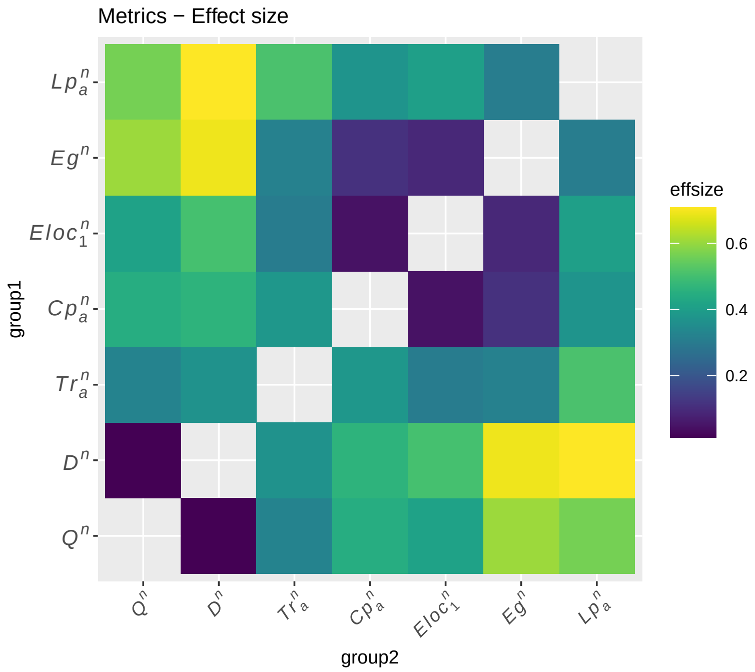

Fig. S36. Metrics - Effect size

Table S37. Metrics - Effect size

character1	character2	effsize	n1	n2	magnitude
Cp_a^n	$Eloc_1^n$	0.0442149	8064	8064	small
Cp_a^n	Lp_a^n	0.3729141	8064	8064	moderate
Cp_a^n	Eg^n	0.1094179	8064	8064	small
Cp_a^n	Q^n	0.4462449	8064	8064	moderate
Cp_a^n	D^n	0.4629948	8064	8064	moderate
Cp_a^n	Tr_a^n	0.3823045	8064	8064	moderate
$Eloc_1^n$	Lp_a^n	0.4014701	8064	8064	moderate
$Eloc_1^n$	Eg^n	0.0895200	8064	8064	small
$Eloc_1^n$	Q^n	0.4124881	8064	8064	moderate
$Eloc_1^n$	D^n	0.5027420	8064	8064	large
$Eloc_1^n$	Tr_a^n	0.3025436	8064	8064	moderate
Lp_a^n	Eg^n	0.3055529	8064	8064	moderate
Lp_a^n	Q^n	0.5641222	8064	8064	large
Lp_a^n	D^n	0.7079035	8064	8064	large
Lp_a^n	Tr_a^n	0.5101946	8064	8064	large
Eg^n	Q^n	0.6054757	8064	8064	large
Eg^n	D^n	0.6915405	8064	8064	large
Eg^n	Tr_a^n	0.3167091	8064	8064	moderate
Q^n	D^n	0.0132182	8064	8064	small
Q^n	Tr_a^n	0.3249259	8064	8064	moderate
D^n	Tr_a^n	0.3637976	8064	8064	moderate

6 More Metrics - Network Analysis

6.1 ICC Density distribution

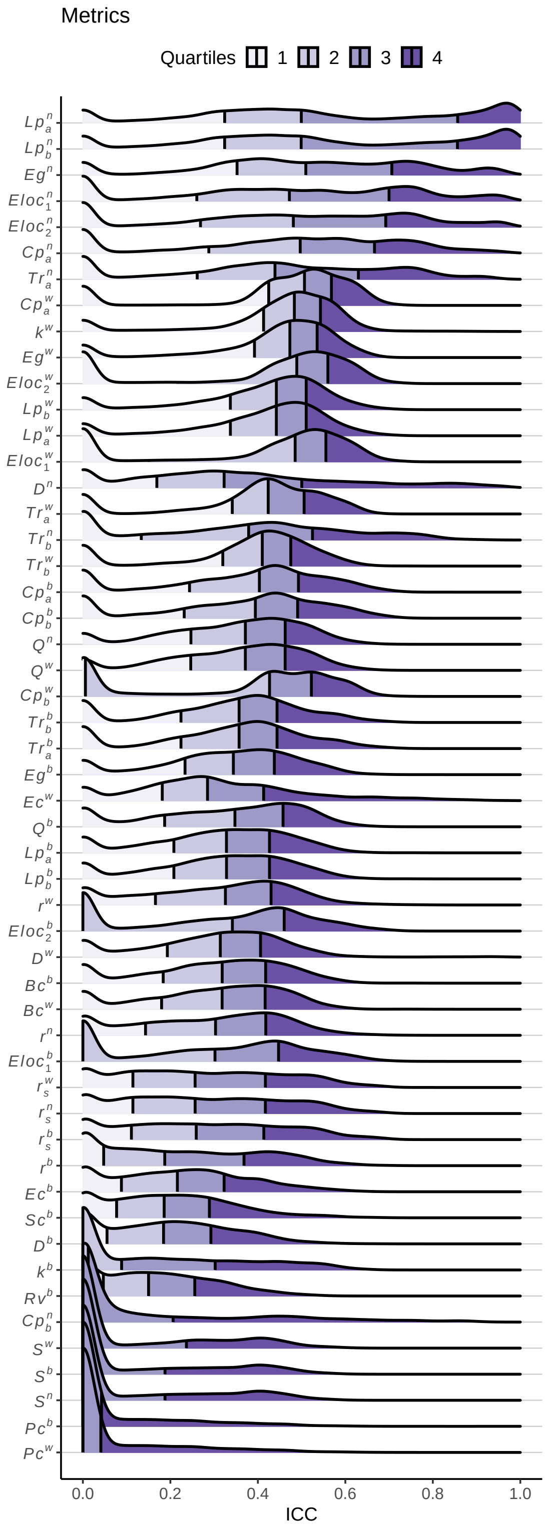

Fig. S37. Metrics - Density distribution

6.2 Almost Perfect (ICCs > 0.8)

Table S38. Metrics - Number of ICCs > 0.8

	character	n
14	Lp_a^n	2350
11	Lp_b^n	2349
16	Eg^n	924
8	$Eloc_1^n$	835
9	$Eloc_2^n$	792
5	Cp_a^n	575
17	D^n	559
21	Tr_a^n	433
6	Ec^w	137
19	Tr_b^n	131
4	Cp_b^n	114
18	D^w	57
15	k^w	27
12	Pc^b	4
1	r^w	2
2	Bc^b	1
3	Bc^w	1
7	$Eloc_1^b$	1
10	Lp_b^b	1
13	Lp_a^b	1
20	Tr_b^w	1

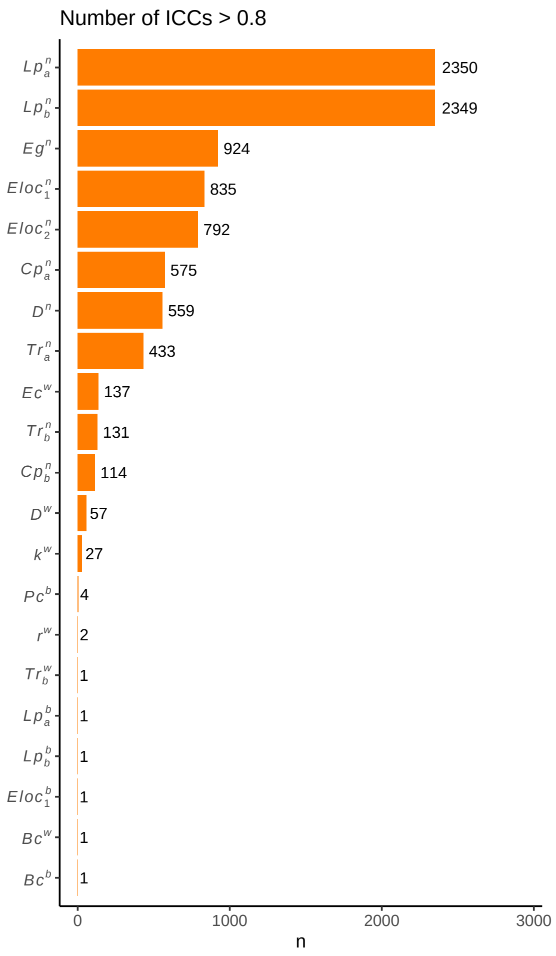

Fig. S38. Metrics - Number of ICC > 0.8

6.3 Substantial or Above (ICCs > 0.6)

Table S39. Metrics - Number of ICCs > 0.6

	character	n
27	Lp_a^n	3194
21	Lp_b^n	3190
33	Eg^n	3032
18	$Eloc_2^n$	2913
15	$Eloc_1^n$	2886
10	Cp_a^n	2709
51	Tr_a^n	2273
39	D^n	1383
48	Tr_b^n	1316
11	Cp_a^w	1302
19	$Eloc_2^w$	1085
16	$Eloc_1^w$	998
13	Ec^w	664

Table S39. Metrics - Number of ICCs > 0.6 *(continued)*

	character	n
9	Cp_a^b	603
6	Cp_b^b	595
8	Cp_b^w	592
31	k^w	481
34	Eg^w	467
7	Cp_b^n	463
52	Tr_a^w	385
22	Lp_b^w	375
28	Lp_a^w	375
17	$Eloc_2^b$	345
14	$Eloc_1^b$	314
47	Tr_b^b	276
50	Tr_a^b	276
41	r_s^b	247
42	r_s^n	229
43	r_s^w	229
49	Tr_b^w	153
3	r^w	148
36	Q^n	140
37	Q^w	140
35	Q^b	109
40	D^w	107
2	r^n	100
32	Eg^b	83
1	r^b	81
20	Lp_b^b	67
26	Lp_a^b	67
30	k^b	64
4	Bc^b	50
12	Ec^b	40
29	Sc^b	40
5	Bc^w	32
23	Pc^b	18
38	D^b	16
24	Pc^w	14
46	S^w	12
25	Rv^b	2
44	S^b	2
45	S^n	2

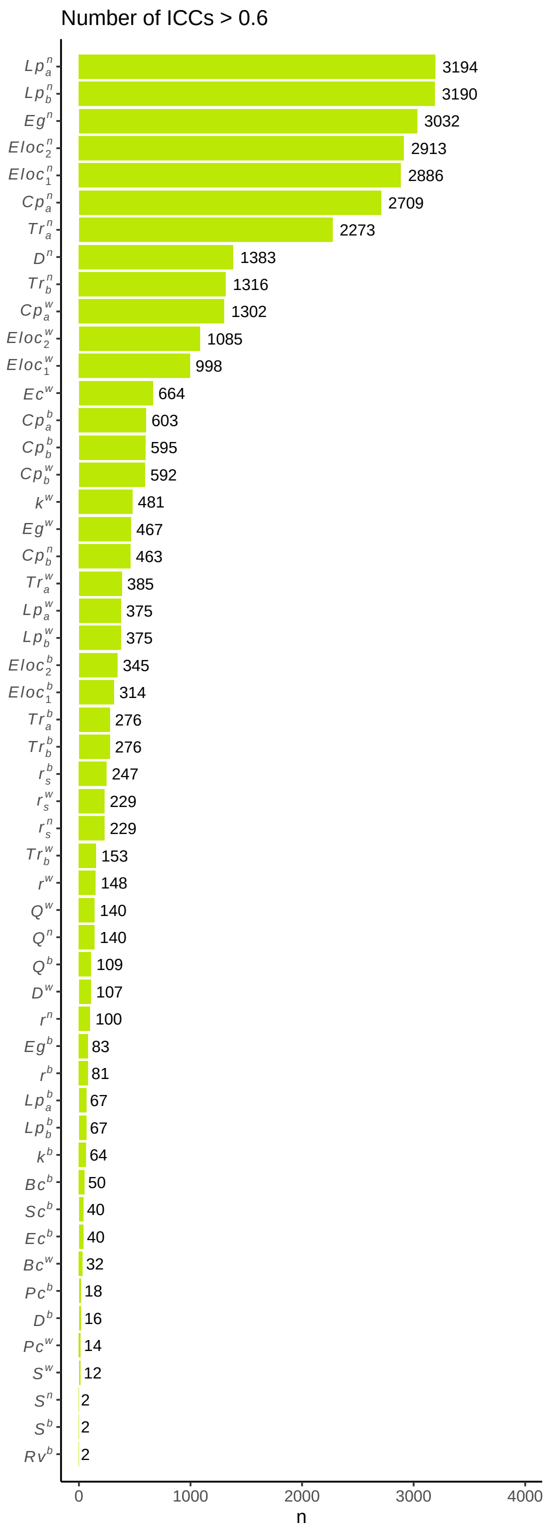

Fig. S39. Metrics - Number of ICC > 0.6

6.4 Descriptive statistics Mean

Table S40. Metrics - ICC Mean

character	n	meanz2r
Lp_a^n	8064	0.6847483
Lp_b^n	8064	0.6842168
Eg^n	8064	0.5628534
$Eloc_1^n$	8064	0.5241176
$Eloc_2^n$	8064	0.5219382
Cp_a^n	8064	0.5057482
Tr_a^n	8064	0.4683854
Cp_a^w	8064	0.4621172
k^w	8064	0.4542164
Eg^w	8064	0.4381993
$Eloc_2^w$	8064	0.4144730
Lp_b^w	8064	0.4094914
Lp_a^w	8064	0.4094914
$Eloc_1^w$	8064	0.4053213
D^n	8064	0.3952442
Tr_a^w	8064	0.3935553
Tr_b^n	8064	0.3790930
Tr_b^w	8064	0.3704978
Cp_a^b	8064	0.3679068
Cp_b^b	8064	0.3627075
Q^n	8064	0.3531167
Q^w	8064	0.3531167
Cp_b^w	8064	0.3487325
Tr_b^b	8064	0.3346006
Tr_a^b	8064	0.3346006
Eg^b	8064	0.3301530
Ec^w	8064	0.3265843
Q^b	8064	0.3239017
Lp_b^b	8064	0.3140209
Lp_a^b	8064	0.3140209
r^w	8064	0.3067918
$Eloc_2^b$	8064	0.3049789
D^w	8064	0.3031638
Bc^b	8064	0.3013466
Bc^w	8064	0.2986165
r^n	8064	0.2885648
$Eloc_1^b$	8064	0.2858122
r_s^n	8064	0.2802930
r_s^w	8064	0.2802930
r_s^b	8064	0.2784491
r^b	8064	0.2231797

Table S40. Metrics - ICC Mean *(continued)*

character	n	meanz2r
Ec^b	8064	0.2222293
Sc^b	8064	0.1983362
D^b	8064	0.1916023
k^b	8064	0.1712945
Rv^b	8064	0.1664371
Cp_b^n	8064	0.1566982
S^w	8064	0.1135087
S^b	8064	0.1036267
S^n	8064	0.1036267
Pc^b	8064	0.0629168
Pc^w	8064	0.0619207

6.5 Descriptive statistics Median

Table S41. Metrics - ICC Median

character	n	median
Eg^n	8064	0.510
Cp_a^w	8064	0.507
Lp_b^n	8064	0.499
Lp_a^n	8064	0.499
Cp_a^n	8064	0.497
$Eloc_2^w$	8064	0.489
$Eloc_1^w$	8064	0.485
k^w	8064	0.483
$Eloc_2^n$	8064	0.481
Eg^w	8064	0.473
$Eloc_1^n$	8064	0.472
Lp_b^w	8064	0.442
Lp_a^w	8064	0.442
Tr_a^n	8064	0.439
Cp_b^w	8064	0.427
Tr_a^w	8064	0.424
Tr_b^w	8064	0.410
Cp_a^b	8064	0.403
Cp_b^b	8064	0.394
Tr_b^n	8064	0.379
Q^n	8064	0.371
Q^w	8064	0.371
Tr_b^b	8064	0.357
Tr_a^b	8064	0.357

Table S41. Metrics - ICC Median *(continued)*

character	n	median
Q^b	8064	0.348
Eg^b	8064	0.344
$Eloc_2^b$	8064	0.342
Lp_b^b	8064	0.328
Lp_a^b	8064	0.328
r^w	8064	0.326
D^n	8064	0.323
Bc^b	8064	0.318
Bc^w	8064	0.318
D^w	8064	0.314
r^n	8064	0.303
$Eloc_1^b$	8064	0.302
Ec^w	8064	0.285
r_s^b	8064	0.259
r_s^n	8064	0.257
r_s^w	8064	0.257
Ec^b	8064	0.216
r^b	8064	0.187
Sc^b	8064	0.186
D^b	8064	0.184
Rv^b	8064	0.150
k^b	8064	0.089
Cp_b^n	8064	0.012
Pc^b	8064	0.000
Pc^w	8064	0.000
S^b	8064	0.000
S^n	8064	0.000
S^w	8064	0.000

6.6 Kullback-leibler divergence Map

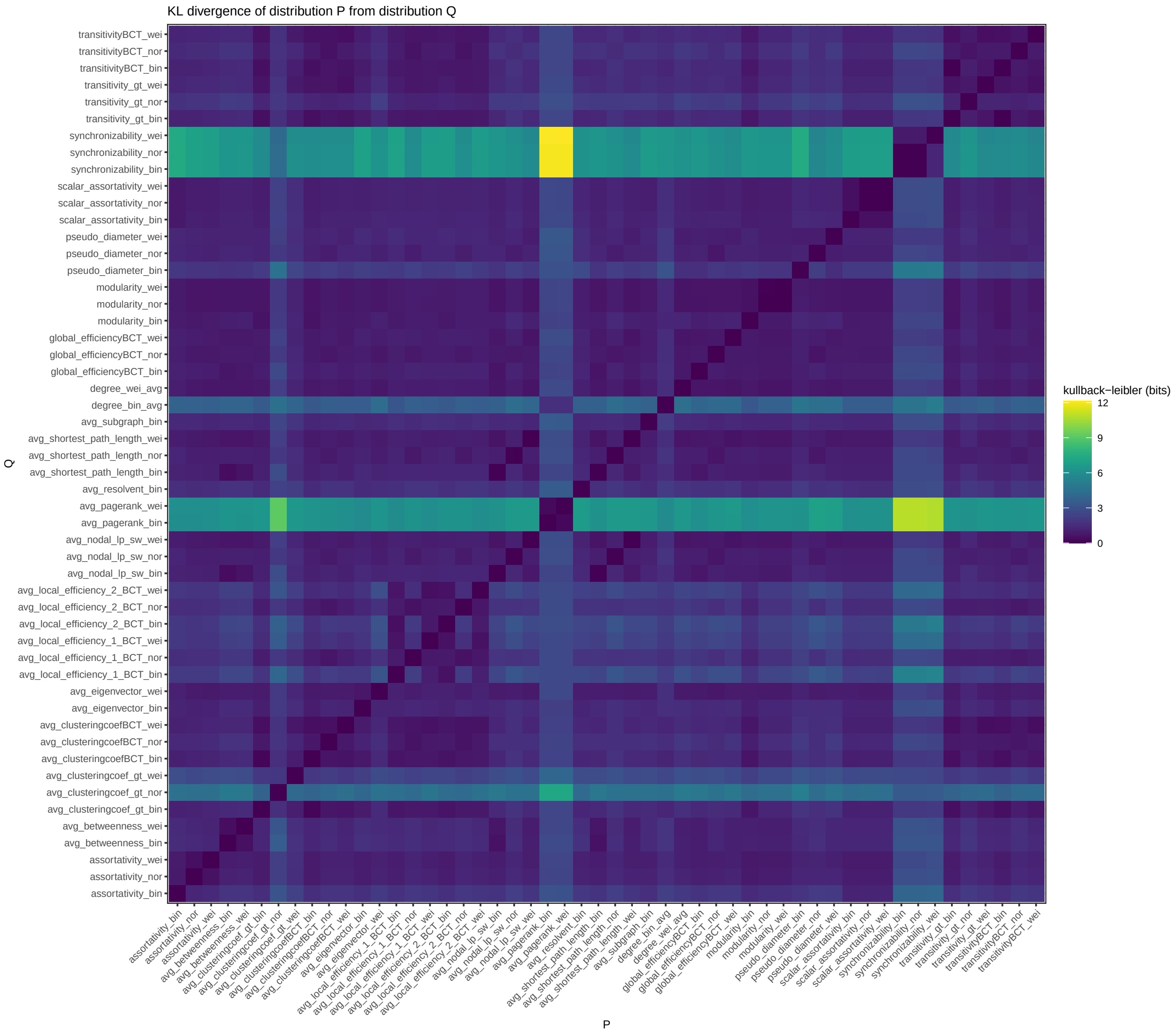

Fig. S40. Metrics - KL divergence Map

6.7 Significance map

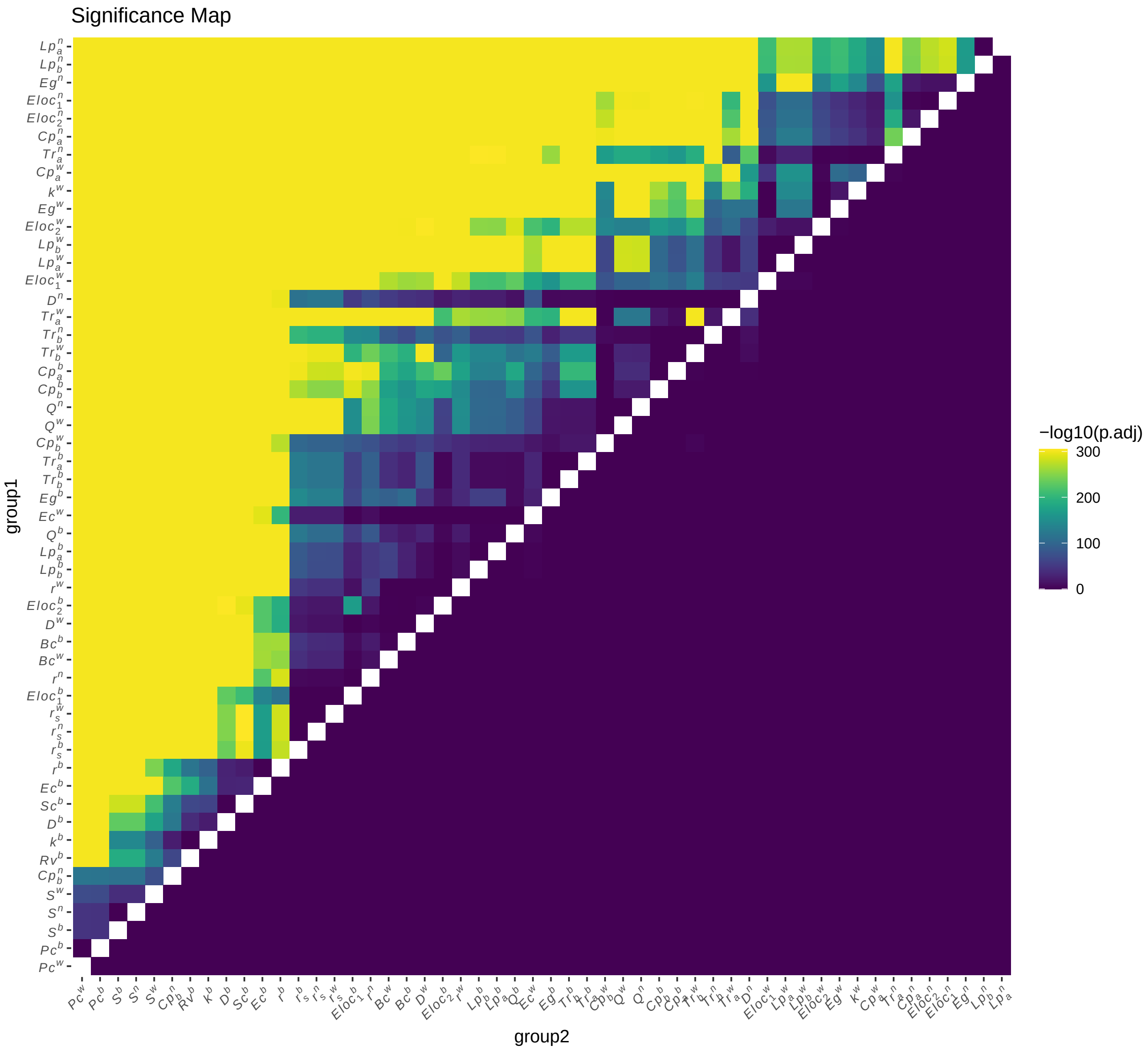

Fig. S41. Metrics - Significance Map

7 fMRIPrep pipeline

We generated the HCP data preprocessed by the fMRI-Prep pipeline and applied our analytical workflow to the preprocessed data. According to the high computational cost and resource intensity of fully computing the results using this pipeline, as well as the large number of conditions derived from the approximately 40+ subjects and the use of threshold-based methods such as GCE, we were only able to study the results generated by the fMRIPrep pipeline to a limited extent but the general patterns of the findings are reproducible.

Our analysis of the fMRIPrep results showed a similar gradient of changes across the frequency bands (Figure S42), but the whole-brain results were not significantly superior to the cortical results. The figure shows that the ICC values generally increase with higher frequency bands (slow6 to slow2, with slow1 not fully shown), consistent with the results in the original study. However, the differences between parcellations are not significant, with no clear advantage of whole brain over cortex, which may be related to the variable

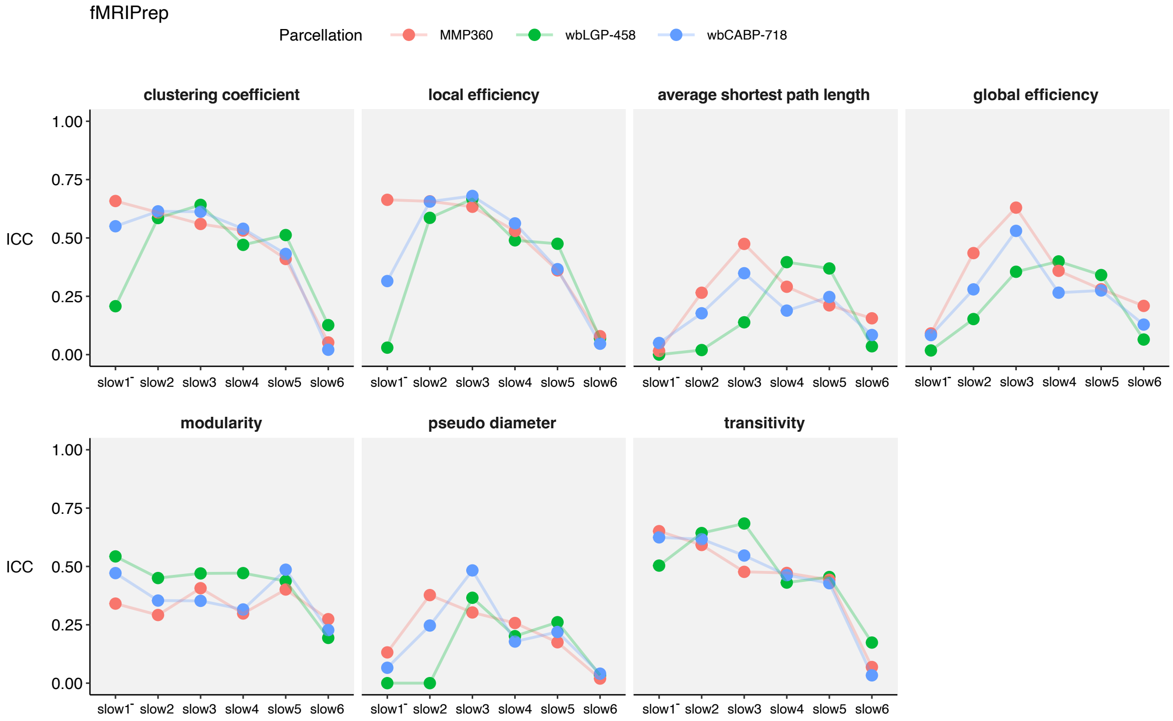

Fig. S42. Test-retest reliability of fMRIPrep-preprocessed data with different frequency bands and parcellations. The ICC values of global graph measures including average shortest path length (L_p), global efficiency (E_g), pseudo diameter (D), clustering coefficient (C_p), local efficiency (E_{loc}), transitivity (Tr), modularity (Q) under sampling conditions (pos and OMST) in different frequency bands (slow6 to slow1) and parcellations (MMP-360, wbLGP-458, wbCABP-718) are plotted. The dots are color-coded according to the parcellations: red for MMP-360, green for wbLGP-458, and blue for wbCABP-718.

pre-processing pipelines. Although the results were not as impressive as those obtained using the HCP pipeline, the overall trend of the results remained consistent. It is worth noting that this study is primarily based on the HCP pipeline, and we have added a new section to the discussion to clarify this point, in which we discuss the potential impact of different preprocessing choices on the reliability of functional connectivity measures, and the importance of carefully considering these choices in future studies. We will continue to consider the fMRIPrep pipeline in future research, as appropriate. The details of the fMRI-prep analyses are documented below and included as supplementary materials as well as discussed in terms of reproducibility in the revised manuscript.

The figure S43 compares the reliability of graph theory measures and frequency bands using fMRIPrep preprocessing, following the parameterization of the optimal pipeline described in the original manuscript. As in Figure 3a, we decomposed the reliability of different network metrics onto the reliability anatomy plane, which plots reliability as a function of between-subject variability (V_b) and within-subject variability (V_w). Comparison to the results obtained using the HCP preprocessing pipeline (Figure 5 in the main text) shows that segregation measures have similar levels of reliability for both preprocessing pipelines, while integration measures are significantly lower for the fMRIPrep pipeline. The reliability of different frequency bands onto the reliability anatomy plane shows a trend of increasing reliability from slow-6 to slow-2 bands (Figure 3b), similar to the HCP pipeline. However, slow-1 band exhibits lower reliability, likely due to the partial coverage of the slow-1 frequency range. Overall, the results suggest that fMRIPrep preprocessing produces similar trends in reliability as the HCP pipeline, but with generally lower overall reliability for both network metrics and frequency bands.

We note that the fMRIPrep preprocessing pipeline was performed using *fMRIPrep* 21.0.2 (Esteban,

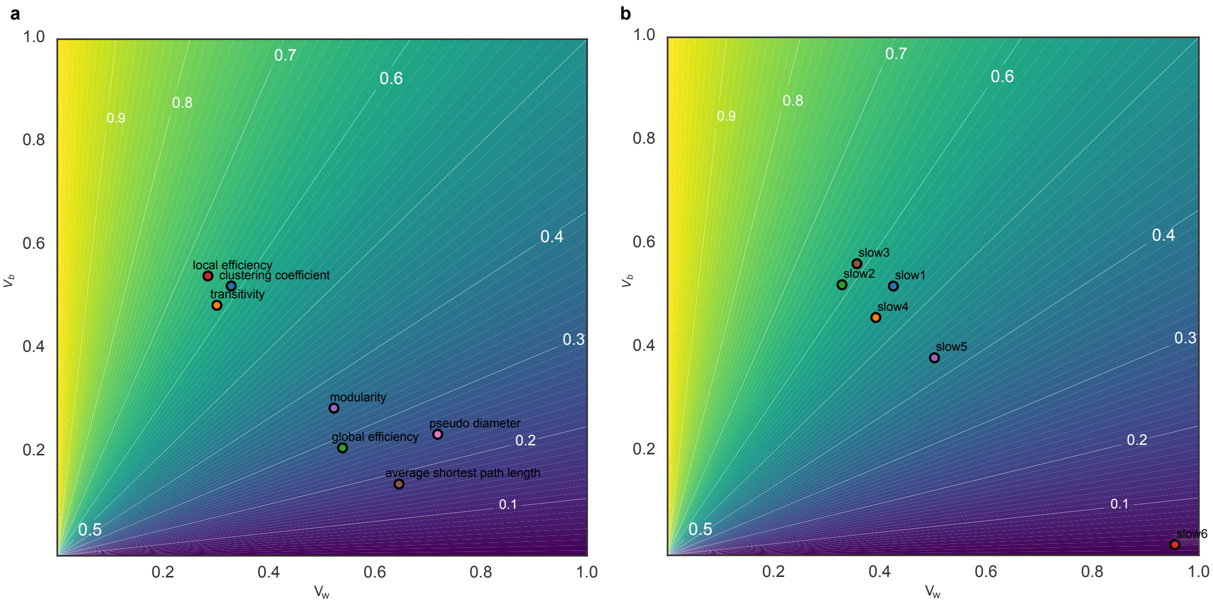

Fig. S43. Reliability across different network measures and frequency bands using fMRIPrep. (a) Reliability anatomy of global network measures. The reliability anatomy was plotted as a function of between-subject variability (V_b) and within-subject variability (V_w). (b) Reliability anatomy of frequency bands.

Markiewicz, et al. (2018); Esteban, Blair, et al. (2018); RRID:SCR_016216), which is based on *Nipype* 1.6.1 (K. Gorgolewski et al. (2011); K. J. Gorgolewski et al. (2018); RRID:SCR_002502). We performed additional standard preprocessing steps on the fmriprep output dtseries data. This included demeaning and detrending the time series, and performing nuisance regression on the minimally preprocessed data. Nuisance regression was based on empirical validation tests by Ciric et al. (2017) to reduce the effects of motion and physiological noise. Specifically, six primary motion parameters were removed, along with their derivatives, and the quadratics of all regressors (24 motion regressors in total). Physiologic noise was modeled using aCompCor on time series extracted from the white matter and ventricles (Behzadi, Restom, Liau, & Liu, 2007a). For aCompCor, the first five principal components from the white matter and ventricles were extracted separately and included in the nuisance regression. In addition, we included the derivatives of each of those components, and the quadratics of all physiological noise regressors (40 physiological noise regressors in total). The nuisance regression model contained a total of 64 nuisance parameters. Note that aCompCor was used in place of global signal regression, given evidence that it has similar benefits as global signal regression for removing artifacts (Power et al., 2018) but without regressing gray matter signals (mixed with other gray matter signals) from themselves, which may result in false correlations (Murphy, Birn, Handwerker, Jones, & Bandettini, 2009; Power, Laumann, Plitt, Martin, & Petersen, 2017).

Then, the cleaned data was fed into our previous ICC optimization pipeline. In order to further verify the results of our previous ICC optimization pipeline, we expanded our analyses to include additional parcellations (MMP-360, wbLGP-458, wbCABP-718) and frequency bands (slow6 to slow1). These additional analyses were performed using the same main calculation conditions as our previous optimal pipeline (wbLGP-458, slow2, OMST, pos, normalization weighted). However, due to considerations of computational complexity and resources, we were unable to completely replicate all of the conditions from our previous analyses and instead only included a subset of the data. As a result, we were unable to generate distribution plots similar to those in our previous analyses, and instead only present discrete points for these additional ICC values. Despite this, we were still able to decompose these ICC values in terms of between- and within-subject variability. Through these additional analyses, we were able to gain some insight into the impact of using the fmriprep preprocessing pipeline on ICC values.

Preprocessing of B0 inhomogeneity mappings A total of 3 fieldmaps were found available within the

input BIDS structure for this particular subject. A *B0* nonuniformity map (or *fieldmap*) was estimated from the phase-drift map(s) measure with two consecutive GRE (gradient-recalled echo) acquisitions. The corresponding phase-map(s) were phase-unwrapped with `prelude` (FSL 6.0.5.1:57b01774). A *B0*-nonuniformity map (or *fieldmap*) was estimated based on two (or more) echo-planar imaging (EPI) references with `topup` (Andersson, Skare, and Ashburner (2003); FSL 6.0.5.1:57b01774).

Anatomical data preprocessing A total of 1 T1-weighted (T1w) images were found within the input BIDS dataset.The T1-weighted (T1w) image was corrected for intensity non-uniformity (INU) with `N4BiasFieldCorrection` (Tustison et al., 2010), distributed with ANTs 2.3.3 (Avants, Epstein, Grossman, & Gee, 2008, RRID:SCR_004757), and used as T1w-reference throughout the workflow. The T1w-reference was then skull-stripped with a *Nipype* implementation of the workflow (from ANTs), using OASIS30ANTs as target template. Brain tissue segmentation of cerebrospinal fluid (CSF), white-matter (WM) and gray-matter (GM) was performed on the brain-extracted T1w using `fast` (FSL 6.0.5.1:57b01774, RRID:SCR_002823, Zhang, Brady, & Smith, 2001). Brain surfaces were reconstructed using `recon-all` (FreeSurfer 6.0.1, RRID:SCR_001847, Dale, Fischl, & Sereno, 1999), and the brain mask estimated previously was refined with a custom variation of the method to reconcile ANTs-derived and FreeSurfer-derived segmentations of the cortical gray-matter of Mindboggle (RRID:SCR_002438, Klein et al., 2017). Volume-based spatial normalization to two standard spaces (MNI152NLin2009cAsym, MNI152NLin6Asym) was performed through nonlinear registration with `antsRegistration` (ANTs 2.3.3), using brain-extracted versions of both T1w reference and the T1w template. The following templates were selected for spatial normalization: *ICBM 152 Nonlinear Asymmetrical template version 2009c* [Fonov, Evans, McKinstry, Almli, and Collins (2009), RRID:SCR_008796; TemplateFlow ID: MNI152NLin2009cAsym], *FSL's MNI ICBM 152 non-linear 6th Generation Asymmetric Average Brain Stereotaxic Registration Model* [Evans, Janke, Collins, and Baillet (2012), RRID:SCR_002823; TemplateFlow ID: MNI152NLin6Asym].

Functional data preprocessing For each of the 4 BOLD runs found per subject (across all tasks and sessions), the following preprocessing was performed. First, a reference volume and its skull-stripped version were generated by aligning and averaging 1 single-band references (SBRefs). Head-motion parameters with respect to the BOLD reference (transformation matrices, and six corresponding rotation and translation parameters) are estimated before any spatiotemporal filtering using `mcflirt` (FSL 6.0.5.1:57b01774, Jenkinson, Bannister, Brady, & Smith, 2002). The estimated *fieldmap* was then aligned with rigid-registration to the target EPI (echo-planar imaging) reference run. The field coefficients were mapped on to the reference EPI using the transform. The BOLD reference was then co-registered to the T1w reference using `bbregister` (FreeSurfer) which implements boundary-based registration (Greve & Fischl, 2009). Co-registration was configured with six degrees of freedom. First, a reference volume and its skull-stripped version were generated using a custom methodology of *fMRIPrep*. Several confounding time-series were calculated based on the *preprocessed BOLD*: framewise displacement (FD), DVARS and three region-wise global signals. FD was computed using two formulations following Power (absolute sum of relative motions, Power et al. (2014)) and Jenkinson (relative root mean square displacement between affines, Jenkinson et al. (2002)). FD and DVARS are calculated for each functional run, both using their implementations in *Nipype* (following the definitions by Power et al., 2014). The three global signals are extracted within the CSF, the WM, and the whole-brain masks. Additionally, a set of physiological regressors were extracted to allow for component-based noise correction (*CompCor*, Behzadi, Restom, Liau, & Liu, 2007b). Principal components are estimated after high-pass filtering the *preprocessed BOLD* time-series (using a discrete cosine filter with 128s cut-off) for the two *CompCor* variants: temporal (tCompCor) and anatomical (aCompCor). tCompCor components are then calculated from the top 2% variable voxels within the brain mask. For aCompCor, three probabilistic masks (CSF, WM and combined CSF+WM) are generated in anatomical space. The implementation differs from that of Behzadi et al. in that instead of eroding the masks by 2 pixels on BOLD space, the aCompCor masks are subtracted a mask of pixels that likely contain a volume fraction of GM. This mask is obtained by dilating a GM mask extracted from the FreeSurfer's *aseg* segmentation, and it ensures components are not extracted from voxels containing a minimal fraction of GM. Finally, these masks are resampled into BOLD space and binarized by thresholding at 0.99 (as in the original implementation). Components are also calculated separately

within the WM and CSF masks. For each CompCor decomposition, the *k* components with the largest singular values are retained, such that the retained components' time series are sufficient to explain 50 percent of variance across the nuisance mask (CSF, WM, combined, or temporal). The remaining components are dropped from consideration. The head-motion estimates calculated in the correction step were also placed within the corresponding confounds file. The confound time series derived from head motion estimates and global signals were expanded with the inclusion of temporal derivatives and quadratic terms for each (Satterthwaite et al., 2013). Frames that exceeded a threshold of 0.5 mm FD or 1.5 standardised DVARS were annotated as motion outliers. The BOLD time-series were resampled into standard space, generating a *preprocessed BOLD run in MNI152NLin2009cAsym space*. First, a reference volume and its skull-stripped version were generated using a custom methodology of *fMRIPrep*. The BOLD time-series were resampled onto the following surfaces (FreeSurfer reconstruction nomenclature): *fsaverage*. Automatic removal of motion artifacts using independent component analysis (ICA-AROMA, Pruim et al., 2015) was performed on the *preprocessed BOLD on MNI space* time-series after removal of non-steady state volumes and spatial smoothing with an isotropic, Gaussian kernel of 6mm FWHM (full-width half-maximum). Corresponding "non-aggresively" denoised runs were produced after such smoothing. Additionally, the "aggressive" noise-regressors were collected and placed in the corresponding confounds file. *Grayordinates* files (Glasser et al., 2013) containing 91k samples were also generated using the highest-resolution `fsaverage` as intermediate standardized surface space. All resamplings can be performed with *a single interpolation step* by composing all the pertinent transformations (i.e. head-motion transform matrices, susceptibility distortion correction when available, and co-registrations to anatomical and output spaces). Gridded (volumetric) resamplings were performed using `antsApplyTransforms` (ANTs), configured with Lanczos interpolation to minimize the smoothing effects of other kernels (Lanczos, 1964). Non-gridded (surface) resamplings were performed using `mri_vol2surf` (FreeSurfer). Many internal operations of *fMRIPrep* use *Nilearn* 0.8.1 (Abraham et al., 2014, RRID:SCR_001362), mostly within the functional processing workflow. For more details of the pipeline, see the section corresponding to workflows in *fMRIPrep*'s documentation.

References

Abraham, A., Pedregosa, F., Eickenberg, M., Gervais, P., Mueller, A., Kossaifi, J., … Varoquaux, G. (2014). Machine learning for neuroimaging with scikit-learn. *Frontiers in Neuroinformatics*, *8*.

Andersson, J. L., Skare, S., & Ashburner, J. (2003). How to correct susceptibility distortions in spin-echo echo-planar images: application to diffusion tensor imaging. *NeuroImage*, *20*(2), 870-888.

Avants, B., Epstein, C., Grossman, M., & Gee, J. (2008). Symmetric diffeomorphic image registration with cross-correlation: Evaluating automated labeling of elderly and neurodegenerative brain. *Medical Image Analysis*, *12*(1), 26-41.

Behzadi, Y., Restom, K., Liau, J., & Liu, T. T. (2007a). A component based noise correction method (compcor) for bold and perfusion based fmri. *Neuroimage*, *37*(1), 90–101.

Behzadi, Y., Restom, K., Liau, J., & Liu, T. T. (2007b). A component based noise correction method (CompCor) for BOLD and perfusion based fmri. *NeuroImage*, *37*(1), 90-101.

Ciric, R., Wolf, D. H., Power, J. D., Roalf, D. R., Baum, G. L., Ruparel, K., … others (2017). Benchmarking of participant-level confound regression strategies for the control of motion artifact in studies of functional connectivity. *Neuroimage*, *154*, 174–187.

Dale, A. M., Fischl, B., & Sereno, M. I. (1999). Cortical surface-based analysis: I. segmentation and surface reconstruction. *NeuroImage*, *9*(2), 179-194.

Esteban, O., Blair, R., Markiewicz, C. J., Berleant, S. L., Moodie, C., Ma, F., … Gorgolewski, K. J. (2018). fmriprep. *Software*.

Esteban, O., Markiewicz, C., Blair, R. W., Moodie, C., Isik, A. I., Erramuzpe Aliaga, A., … Gorgolewski, K. J. (2018). fMRIPrep: a robust preprocessing pipeline for functional MRI. *Nature Methods*.

Evans, A., Janke, A., Collins, D., & Baillet, S. (2012). Brain templates and atlases. *NeuroImage*, *62*(2), 911–922.

Fonov, V., Evans, A., McKinstry, R., Almli, C., & Collins, D. (2009). Unbiased nonlinear average age-appropriate brain templates from birth to adulthood. *NeuroImage*, *47, Supplement 1*, S102.

Glasser, M. F., Sotiropoulos, S. N., Wilson, J. A., Coalson, T. S., Fischl, B., Andersson, J. L., … Jenkinson, M. (2013). The minimal preprocessing pipelines for the human connectome project. *NeuroImage*, *80*, 105-124.

Gorgolewski, K., Burns, C. D., Madison, C., Clark, D., Halchenko, Y. O., Waskom, M. L., & Ghosh, S. (2011). Nipype: a flexible, lightweight and extensible neuroimaging data processing framework in python. *Frontiers in Neuroinformatics*, *5*, 13.

Gorgolewski, K. J., Esteban, O., Markiewicz, C. J., Ziegler, E., Ellis, D. G., Notter, M. P., … Ghosh, S. (2018). Nipype. *Software*.

Greve, D. N., & Fischl, B. (2009). Accurate and robust brain image alignment using boundary-based registration. *NeuroImage*, *48*(1), 63-72.

Jenkinson, M., Bannister, P., Brady, M., & Smith, S. (2002). Improved optimization for the robust and accurate linear registration and motion correction of brain images. *NeuroImage*, *17*(2), 825-841.

Klein, A., Ghosh, S. S., Bao, F. S., Giard, J., Häme, Y., Stavsky, E., … Keshavan, A. (2017). Mindboggling morphometry of human brains. *PLOS Computational Biology*, *13*(2), e1005350.

Lanczos, C. (1964). Evaluation of noisy data. *Journal of the Society for Industrial and Applied Mathematics Series B Numerical Analysis*, *1*(1), 76-85.

Murphy, K., Birn, R. M., Handwerker, D. A., Jones, T. B., & Bandettini, P. A. (2009). The impact of global signal regression on resting state correlations: are anti-correlated networks introduced? *Neuroimage*, *44*(3), 893–905.

Power, J. D., Laumann, T. O., Plitt, M., Martin, A., & Petersen, S. E. (2017). On global fmri signals and simulations. *Trends in cognitive sciences*, *21*(12), 911–913.

Power, J. D., Mitra, A., Laumann, T. O., Snyder, A. Z., Schlaggar, B. L., & Petersen, S. E. (2014). Methods to detect, characterize, and remove motion artifact in resting state fmri. *NeuroImage*, *84*(Supplement C), 320-341.

Power, J. D., Plitt, M., Gotts, S. J., Kundu, P., Voon, V., Bandettini, P. A., & Martin, A. (2018). Ridding fmri data of motion-related influences: Removal of signals with distinct spatial and physical bases in multiecho data. *Proceedings of the National Academy of Sciences*, *115*(9), E2105–E2114.

Pruim, R. H. R., Mennes, M., van Rooij, D., Llera, A., Buitelaar, J. K., & Beckmann, C. F. (2015). Ica-AROMA: A robust ICA-based strategy for removing motion artifacts from fmri data. *NeuroImage*, *112*(Supplement C), 267-277.

Satterthwaite, T. D., Elliott, M. A., Gerraty, R. T., Ruparel, K., Loughead, J., Calkins, M. E., … Wolf, D. H. (2013). An improved framework for confound regression and filtering for control of motion artifact in the preprocessing of resting-state functional connectivity data. *NeuroImage*, *64*(1), 240–256.

Tustison, N. J., Avants, B. B., Cook, P. A., Zheng, Y., Egan, A., Yushkevich, P. A., & Gee, J. C. (2010). N4itk: Improved n3 bias correction. *IEEE Transactions on Medical Imaging*, *29*(6), 1310-1320.

Zhang, Y., Brady, M., & Smith, S. (2001). Segmentation of brain MR images through a hidden markov random field model and the expectation-maximization algorithm. *IEEE Transactions on Medical Imaging*, *20*(1), 45-57.
